# Beyond BRCA deficiency: Clinical and molecular predictors of survival in patients with BRCA-deficient tubo-ovarian high-grade serous carcinoma

**DOI:** 10.21203/rs.3.rs-7572112/v1

**Published:** 2025-10-03

**Authors:** Dale Garsed, Tibor Zwimpfer, Sian Fereday, Ahwan Pandey, Dinuka Ariyaratne, Madawa Jayawardana, Laura Twomey, Céline Laumont, Catherine Kennedy, Adelyn Bolithon, Nicola Meagher, Katy Milne, Phineas Hamilton, Jennifer Alsop, Antonis Antoniou, George Au-Yeung, Matthias Beckmann, Amy Berrington de Gonzalez, Christiani Bisinotto, Freya Blome, Clara Bodelon, Jessica Boros, Alison Brand, Michael Carney, Alicia Cazorla-Jimenez, Derek Chiu, Elizabeth Christie, Anita Chudecka-Glaz, Penny Coulson, Kara Cushing-Haugen, Cezary Cybulski, Kathleen Darcy, Cath David, Trent Davidson, Arif Ekici, Esther Elishaev, Julius Emons, Tobias Engler, Rhonda Farrell, Anna Fischer, Montserrat Garcia-Closas, Aleksandra Gentry-Maharaj, Prafull Ghatage, Rosalind Glasspool, Philipp Harter, Andreas Hartkopf, Arndt Hartmann, Sebastian Heikaus, Brenda Hernandez, Anusha Hettiaratchi, Sabine Heublein, David Huntsman, Mercedes Jimenez-Linan, Michael Jones, Eunjoung Kang, Ewa Kaznowska, Tomasz Kluz, Felix Kommoss, Gottfried E. Konecny, Rutgerus Kruitwagen, Jessica Kwon, Diether Lambrechts, Cheng-Han Lee, Jenny Lester, Samuel Leung, Yee Leung, Anna Linder, Jolanta Lissowska, Liselore Loverix, Jan Lubiński, Constantina Mateoiu, lain McNeish, Malak Moubarak, Gregg Nelson, Nikilyn Nevins, Alexander Olawaiye, Siel Olbrecht, Sandra Orsulic, Ana Osorio, Carmel Quinn, Ganendra Raj Mohan, Isabelle Ray-Coquard, Cristina Rodriguez-Antona, Patricia Roxburgh, Matthias Rübner, Stuart Salfinger, Spinder Samra, Minouk Schoemaker, Hans-Peter Sinn, Gabe Sonke, Linda Steele, Colin Stewart, Aline Talhouk, Adeline Tan, Christopher Tarney, Sarah Taylor, Koen Van de Vijver, Maaike Avan der Aa, Toon Van Gorp, Els Van Nieuwenhuysen, Lilian van Wagensveld, Andrea Wahner-Hendrickson, Christina Walter, Chen Wang, Julia Welz, Nicolas Wentzensen, Lynne Wilkens, Stacey Winham, Boris Winterhoff, Michael Anglesio, Andrew Berchuck, Francisco Candido do Reis, Paul Cohen, Thomas Conrads, Philip Crowe, Jennifer Doherty, Peter Fasching, Renée Fortner, Maria Garcia, Simon Gayther, Marc Goodman, Jacek Gronwald, Holly Harris, Florian Heitz, Hugo Horlings, Beth Karlan, Linda Kelemen, George Maxwell, Usha Menon, Francesmary Modugno, Susan Neuhausen, Joellen Schildkraut, Annette Staebler, Karin Sundfeldt, Anthony Swedlow, Ignace Vergote, Anna Wu, James Brenton, Paul Pharoah, Celeste Pearce, Malcolm Pike, Ellen Goode, Susan Ramus, Martin Köbel, Brad Nelson, Anna DeFazio, Michael Friedlander, David Bowtell

**Affiliations:** Peter MacCallum Cancer Centre; Peter MacCallum Cancer Centre; Peter MacCallum Cancer Centre; Peter MacCallum Cancer Centre; Peter MacCallum Cancer Centre; Peter MacCallum Cancer Centre; Peter MacCallum Cancer Centre; Deeley Research Centre, BC Cancer; School of Clinical Medicine, UNSW Medicine and Health, University of NSW Sydney; Adult Cancer Program, Lowy Cancer Research Centre, University of NSW Sydney; University of British Columbia; BC Cancer; Department of Oncology, University of Cambridge; University of Cambridge; Peter MacCallum Cancer Centre; University Breast Center University Hospital Erlangen; National Cancer Institute, NIH, DHHS; Department of Gynecology and Obstetrics, Ribeirão Preto Medical School, University of São Paulo; Institute of Pathology and Neuropathology, Tuebingen University Hospital; Department of Population Science, American Cancer Society, Atlanta; Centre for Cancer Research, The Westmead Institute for Medical Research, Sydney; Westmead Hospital; University of Hawaii, Cancer Research Center; Pathology Department, Fundación Jiménez Díaz. Madrid; University of British Columbia; Peter MacCallum Cancer Centre; Department of Gynecological Surgery and Gynecological Oncology of Adults and Adolescents, Pomeranian Medical University; Division of Genetics and Epidemiology, The Institute of Cancer Research, London; Program in Epidemiology, Division of Public Health Sciences, Fred Hutchinson Cancer Center; Pomeranian Medical School; Gynecologic Cancer Center of Excellence, Department of Gynecologic Surgery and Obstetrics, Uniformed Services University of the Health Sciences, Walter Reed National Military Medical Center; Gynaecological Cancer Centre, Royal Hospital for Women, Randwick; NSW Health Pathology, Prince of Wales Hospital. Sydney; FAU-Uniklinikum Erlangen; Department of Pathology, University of Pittsburgh School of Medicine; Department of Gynecology and Obstetrics, Comprehensive Cancer Center Erlangen-EMN, Friedrich-Alexander University Erlangen-Nuremberg. University Hospital Erlangen; Department of Women’s Health, Tuebingen University Hospital; Faculty of Medicine and Health, The University of Sydney; Institute of Pathology and Neuropathology, Tuebingen University Hospital; National Cancer Institute; UCL EGA Institute for Women’s Health; Department of Oncology, Division of Gynecologic Oncology, Cumming School of Medicine, University of Calgary; Beatson West of Scotland Cancer Centre and School of Cancer Sciences, University of Glasgow; Department of Gynecology and Gynecologic Oncology, Evangelische Kliniken Essen-Mitte; Department of Women’s Health, Tuebingen University Hospital; FAU-Uniklinikum Erlangen; Center for Pathology, Evangelische Kliniken Essen-Mitte; University of Hawai’i Cancer Center; The Health Precincts Biobank, UNSW Biospecimen Services, Mark Wainwright Analytical Centre; Department of Obstetrics and Gynecology, University Hospital Heidelberg; The University of British Columbia; Department of Histopathology, Addenbrooke’s Hospital, Cambridge; Department of Surgery, Seoul National University Bundang Hospital; Department of Pathology, Institute of Medical Sciences, Medical College of Rzeszow University; Department of Gynecology, Gynecology Oncology and Obstetrics, Institute of Medical Sciences, Medical College of Rzeszów University; University Hospital Heidelberg; University of California at Los Angeles; GROW, School for Oncology and Developmental Biology, Maastricht university, 6229 ER Maastricht; Department of Obstetrics and Gynecology, University of British Columbia; KU Leuven & VIB; University of Alberta; University of California at Los Angeles; British Columbia’s Gynecological Cancer Research Team OVCARE, University of British Columbia, BC Cancer, and Vancouver General Hospital; Department of Gynaecological Oncology, King Edward Memorial Hospital, Subiaco; Department of Obstetrics and Gynecology, Institute of Clinical Science, Sahlgrenska Center for Cancer Research, University of Gothenburg; Department of Cancer Epidemiology and Prevention, M Sklodowska-Curie National Research Oncology Institute; Division of Gynecologic Oncology, Department of Gynecology and Obstetrics. Leuven Cancer Institute; Pomeranian Medical University; Department of Pathology, University of Gothenburg; Imperial College London; Department of Gynecology and Gynecologic Oncology, Evangelische Kliniken Essen-Mitte; Department of Oncology, Division of Gynecologic Oncology, Cumming School of Medicine, University of Calgary; Centre for Cancer Research, The Westmead Institute for Medical Research, Sydney; Division of Gynecologic Oncology, Department of Obstetrics, Gynecology and Reproductive Sciences, University of Pittsburgh School of Medicine; Division of Gynecologic Oncology, Department of Gynecology and Obstetrics. Leuven Cancer Institute; David Geffen School of Medicine, Department of Obstetrics and Gynecology, University of California at Los Angeles; Genetics Service, Fundación Jiménez Díaz University Hospital and Health Research Institute, Universidad Autónoma de Madrid IIS-FJD; The Health Precincts Biobank, UNSW Biospecimen Services, Mark Wainwright Analytical Centre; Department of Gynaecological Oncology, King Edward Memorial Hospital, Subiaco; Centre Leon Bérard; Centre for Biomedical Network Research on Rare Diseases CIBERER, Instituto de Salud Carlos III; School of Cancer Sciences, Wolfson Wohl Cancer Research Centre, University of Glasgow; Friedrich-Alexander University of Erlangen-Nuremberg; Department of Gynaecological Oncology, St John of God Subiaco Hospital, Subiaco; Centre for Cancer Research, The Westmead Institute for Medical Research, Sydney; Division of Genetics and Epidemiology, The Institute of Cancer Research, London; Institute of Pathology, Heidelberg University Hospital; The Netherlands Cancer Institute; Beckman Research Institute of City of Hope; King Edward Memorial Hospital; British Columbia’s Gynecological Cancer Research Team OVCARE, University of British Columbia, BC Cancer, and Vancouver General Hospital; Division of Obstetrics and Gynaecology, Medical School, University of Western Australia; Gynecologic Cancer Center of Excellence, Department of Gynecologic Surgery and Obstetrics, Uniformed Services University of the Health Sciences, Walter Reed National Military Medical Center; Division of Gynecologic Oncology, Department of Obstetrics, Gynecology and Reproductive Sciences, University of Pittsburgh School of Medicine; Ghent University Hospital; Department of Research and Development, Netherlands Comprehensive Cancer Organization (IKNL), 3511 DT Utrecht; Division of Gynaecological Oncology, Leuven Cancer Institute, University Hospital Leuven and KU Leuven; University Hospitals Leuven; The Netherlands Cancer Institute; Department of Oncology, Mayo Clinic; Department of Women’s Health, Tuebingen University Hospital; Mayo Clinic; Department of Gynecology and Gynecologic Oncology, Evangelische Kliniken Essen-Mitte; National Cancer Institute; University of Hawaii Cancer Center; Mayo Clinic; Division of Gynecologic Oncology, University of Minnesota, Minneapolis, MN 55455, USA; British Columbia’s Gynecological Cancer Research (OVCARE) Program, University of British Columbia, Vancouver General Hospital, and BC Cancer; Duke University Medical Center; Department of Gynecology and Obstetrics, Ribeirão Preto Medical School, University of São Paulo; Department of Gynaecological Oncology, King Edward Memorial Hospital, Subiaco; Walter Reed National Military Medical Center; School of Clinical Medicine, UNSW Medicine and Health, University of NSW Sydney; Huntsman Cancer Institute, Department of Population Health Sciences, University of Utah; University Hospital Erlangen, Comprehensive Cancer Center Erlangen-EMN, Friedrich Alexander University Erlangen-Nuremberg; Division of Cancer Epidemiology, German Cancer Research Center DKFZ, Heidelberg; Genomic Biomarkers and Precision Oncology Group, Sols-Morreale Biomedical Research Institute IIBM, Consejo Superior de Investigaciones Cientficas & Universidad Autónoma de Madrid CSIC-UAM; Center for Inherited Oncogenesis, Department of Medicine, UT Health San Antonio, San Antonio, TX 78229, USA; CSHS; Pomeranian Medical School; Program in Epidemiology, Division of Public Health Sciences, Fred Hutchinson Cancer Research Center, Seattle, WA; Kliniken Essen-Mitte; The Netherlands Cancer Institute; David Geffen School of Medicine at UCLA; Communicable Disease Epidemiology Section, South Carolina Department of Public Health; Women’s Service Line, Inova Health System; University College London; Division of Gynecologic Oncology, Department of Obstetrics, Gynecology and Reproductive Sciences, University of Pittsburgh School of Medicine; Beckman Research Institute of City of Hope; Department of Epidemiology, Rollins School of Public Health, Emory University; Institute of Pathology and Neuropathology, Tuebingen University Hospital; Department of Obstetrics and Gynecology, Institute of Clinical Science, Sahlgrenska Center for Cancer Research, University of Gothenburg; Division of Genetics and Epidemiology, The Institute of Cancer Research, London; Division of Gynecologic Oncology, Department of Gynecology and Obstetrics. Leuven Cancer Institute; University of Southern California; Cancer Research UK Cambridge Institute, University of Cambridge; Cedars-Sinai Medical Center; University of Michigan-Ann Arbor; mskcc; Mayo Clinic; University of New South Wales Sydney; University of Calgary; University of British Columbia; University of Sydney, Westmead Institute for Medical Research; University of South Wales; Peter Mac Callum Cancer Center

## Abstract

*BRCA*-associated homologous recombination deficiency (HRD) is present in ~ 50% of high-grade serous carcinomas (HGSC) and predicts sensitivity to platinum-based therapy. However, there is little understanding of why some patients with *BRCA*-deficient tumors experience unexpectedly poor outcomes. We profiled 154 tumors, enriched for patients with *BRCA*-deficient tumors that experienced short overall survival (≤ 3 years, n = 42), using whole-genome, transcriptome, and methylation analyses. All but one *BRCA*-deficient tumor exceeded an accepted HRD genomic scarring threshold. However, patients with *BRCA1*-deficient HGSC with a more elevated HRD score survived significantly longer. Patients with *BRCA2*-deficient HGSC and loss of *NF1* survived twice as long as those without *NF1* loss, whereas *PIK3CA* or *RAD21* amplification defined *BRCA2*-deficient HGSC with exceptionally short survival. *BRCA1*-deficient tumors in short survivors had evidence of immunosuppressive c-kit signaling and EMT. In a large HGSC cohort (n = 1,389) including 282 individuals with pathogenic germline *BRCA* variants (g*BRCA*pv), the location of the mutation within functional domains stratified clinical outcomes. Notably, residual disease after primary surgery had limited prognostic effect in g*BRCA*pv-carriers compared to non-carriers. Our findings indicate that tumor HR proficiency in the context of therapy response and survival is not a binary property, and highlight genomic and immune modifiers of outcomes in *BRCA*-deficient HGSC.

## INTRODUCTION

The identification of clinical and molecular determinants of survival in patients with cancer has the dual benefits of finding biomarkers that may guide patient management or provide novel therapeutic opportunities. Until relatively recently, the identification of prognostic biomarkers in ovarian cancer has been confounded by a lack of appreciation of the distinctly different molecular characteristics of the various histologic subtypes that make up epithelial ovarian cancer^1^. Evaluating histologically homogenous sets of ovarian tumors has been critical in deciphering the prognostic importance of proteins such as p53^2,3^ and WT1^4^, and identifying genetic risk loci^5–12^.

High-grade serous carcinoma (HGSC) is the most common histotype, accounting for approximately 70% of ovarian cancer deaths in Western countries^13–16^. Homologous recombination-mediated DNA repair deficiency (HRD) is frequent in HGSC and is most often associated with mutations in *BRCA1* and *BRCA2*^17–19^. Approximately fifty percent of HGSC are regarded to have HRD, a feature that can be inferred through specific patterns of genomic scarring in tumor cells^13,20–25^. HRD leads to genomic instability and tumorigenesis, providing a vulnerability in tumor cells with increased sensitivity to double-strand DNA breaks that can be exploited therapeutically^26–28^. As a result, platinum-based chemotherapy and poly (ADP-ribose) polymerase inhibitor (PARPi) maintenance therapy are generally more effective in patients with HRD tumors^28–33^.

While HRD status is informative, accurate prediction of treatment response and survival in HGSC cannot be simply determined by the presence or absence of mutations in genes associated with HR DNA repair. The initial survival advantage for carriers of pathogenic germline *BRCA1* variants (g*BRCA1*pv) diminishes over time, with fewer g*BRCA1*pv-carriers surviving 10 years after diagnosis than either g*BRCA2*pv-carriers or non-carriers^33–35^. Factors associated with survival outcome in HGSC include residual disease following cytoreductive surgery^16,36–38^, the molecular subtype of the tumor^39^, age at diagnosis^40^, and the extent of T- and B-cell infiltration into tumors^41,42^. In germline pathogenic variant carriers, the location of mutations within *BRCA1* or *BRCA2* or the retention of the wildtype allele in the tumor can result in a hypomorphic phenotype associated with resistance to platinum-based therapy^43–47^. Furthermore, revertant mutations restoring *BRCA1* and *BRCA2* function contribute to acquired resistance to platinum-based therapy and PARPis, impacting treatment response and patient outcomes^48–50^.

Comparing patients who represent the extremes of survival outcomes may provide increased sensitivity to identify prognostic biomarkers that are relevant to a wider patient population^51^. Using this approach, we have recently shown that plasma cell infiltration and other molecular changes, including co-loss of *BRCA* and the tumor suppressor *RB1*, are associated with especially long-term survival in HGSC^22,52,53^. The current study evaluates *BRCA*-deficient HGSC by first focusing on g*BRCA*pv-carriers and then expanding to include somatic mutations and promoter methylation in *BRCA1/2*, and other key HR genes, as well as evaluating tumor HRD status. We focus on patients with either poor or favorable survival outcomes, harnessing the value of analyzing patients with exceptional survival outcomes while comparing cohorts that are as similar as possible in other respects.

## RESULTS

### Association of residual disease with prognosis is attenuated in gBRCApv-carriers

Pathogenic germline *BRCA* variants (g*BRCA*pv) were identified in 20% of patients in the Australian Ovarian Cancer Study (AOCS) cohort (*n* = 282/1389) (Table 1, Supplementary Tables S1 and S2). In applying a survival model, there was evidence that the proportional hazards assumption did not hold (*P* < 0.001), thus an Accelerated Failure Time (AFT) model^54^ was used with results reported as Time Ratios (TR; see Methods), where TR > 1 indicates longer time to progression or death, and a TR < 1 indicates shorter survival or time to progression. Patients with g*BRCA*pvs exhibited improved overall survival (OS; TR: 1.53, 95% CI: 1.33–1.76, *P* < 0.001) and progression-free survival (PFS; TR: 1.34 95% CI: 1.28–1.53, *P* < 0.001) compared with non-carriers (Supplementary Tables S3 and S4).

**Figure 1:**
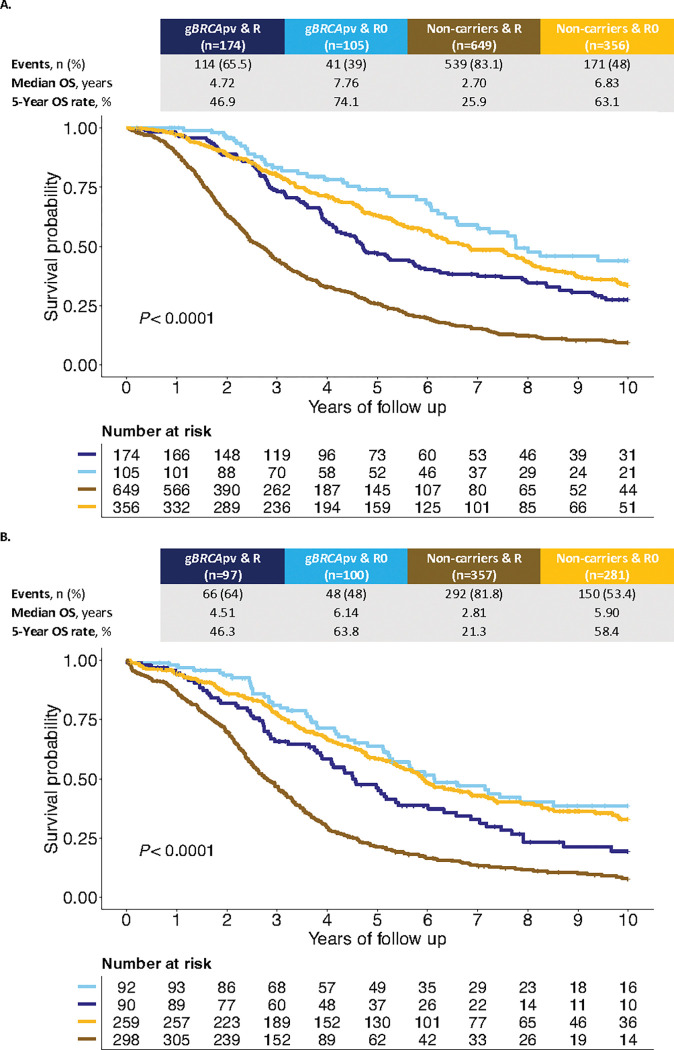
*BRCA* status and residual disease as predictors of overall survival in HGSC. Kaplan-Meier survival curve for the interaction term *BRCA* and Residual status from patients of **a**, the Australian Ovarian Cancer Study (AOCS) cohort and **b**, the Ovarian Tumor Tissue Consortium (OTTA) cohort. *P* values calculated by log-rank test. R=Residual disease, R0=No residual disease, gBRCApv=pathogenic germline BRCA variant, n=Number of patients, OS=Overall survival

We considered whether clinical characteristics differed by germline *BRCA* status and found a statistically significant interaction with residual disease status (*P*-interaction = 0.011; Supplementary Table S3). Using this interaction term, we found that the negative effect of residual disease after cytoreductive surgery on OS was less pronounced in g*BRCA*pv-carriers (TR: 0.87, 95% CI: 0.72–1.06, *P* = 0.162) than in non-carriers (TR: 0.51, 95% CI: 0.44–0.59, *P* < 0.001; Fig. 1a, Table 2). The importance of residual disease for survival in non-carriers was confirmed in the independent OTTA cohort (*n* = 1004, g*BRCA*pv-carriers = 221, 22%; Fig. 1b, Extended Data Figs. 1 and 2a).

We examined the relationship of residual disease and *BRCA* status to known immune and molecular features associated with survival, including tumor-infiltrating lymphocytes (TIL)^42,55^, *RB1* loss^22,52,56^, and transcriptional molecular subtypes^39^. Non-carriers with residual disease had an inverse association with high CD8 + TIL density (*P* = 0.016), with 38.3% of tumors classified as having low or no TIL (Extended Data Fig. 2b, Supplementary Table S5). This group also showed an inverse association with the C4/differentiated (C4.DIF) molecular subtype (*P* = 0.010; Extended Data Fig. 2b). We observed an association between the C1/mesenchymal (C1.MES) molecular subtype and residual disease as previously reported^57^, but this was only statistically significant among non-carriers (*P* = 0.005). *RB1* loss was associated with g*BRCA*pv-carriers without residual disease (*P* < 0.001; Extended Data Fig. 2b).

Although no statistically significant interaction between neoadjuvant chemotherapy (NACT) and *BRCA* status was observed (*P*-interaction = 0.12; Supplementary Table S3), there was evidence of heterogeneity of effect in these subgroups. Among participants who did not receive NACT, g*BRCA*pv-carriers showed a survival benefit compared to non-carriers (TR: 1.60, 95% CI: 1.37–1.87, *P* < 0.001; Supplementary Table S6, Extended Data Fig. 3). In contrast, the overall survival benefit in g*BRCA*pv-carriers versus non-carriers was not statistically significant in the NACT group (TR: 1.39 and 1.17, 95% CI: 0.75–2.60 and 0.62–2.21, *P* = 0.298 and *P* = 0.634 respectively, compared to non-carriers who did not receive NACT).

### gBRCApv location and type are associated with survival and therapy response

Mutations located in various functional domains of *BRCA1* and *BRCA2* have been associated with differences in survival and responses to PARPi in ovarian cancer^43,44^. The mutation type and location of g*BRCA*pvs was ascertained for 240 of the patients in the AOCS cohort from their clinical records and/or previous sequencing analyses^22,56,58,59^ (Extended Data Figs. 4a,b and Supplementary Table S2). Following adjustment for FIGO stage, residual disease status, primary site, age, and first-line treatment, patients with g*BRCA1*pvs in exon 10 had a statistically significant improved OS and PFS (TR: 1.54 and 1.49, 95% CI: 1.19–2.00 and 1.16–1.91, *P* < 0.001 and *P* = 0.002, respectively ), but the association was attenuated for those with variants outside exon 10 (TR: 1.21 and 1.18, 95% CI: 0.97–1.51 and 0.96–1.46, *P* = 0.09 and *P* = 0.12, respectively) compared to non-carriers (Table 3). More specifically, pathogenic variants in the DNA binding domain (DBD) of *BRCA1*, located in exon 10, were associated with an OS and PFS benefit compared to non-carriers (TR: 1.60 and 1.58, 95% CI: 1.14–2.25 and 1.15–2.18, *P* = 0.005 and *P* = 0.006, respectively; Table 3). In contrast, the OS and PFS benefit was not statistically significant for patients with pathogenic variants in the Really Interesting New Gene (RING) (TR: 1.28 and 1.15, 95% CI: 0.87–1.90 and 0.82–1.61, *P* = 0.216 and *P* = 0.419, respectively) and C-terminal domains of *BRCA1* (BRCT) (TR: 1.35 and 1.43, 95% CI: 0.83–2.20 and 0.90–2.26, *P* = 0.222 and *P* = 0.126, respectively), located outside of exon 10.

**Figure 2:**
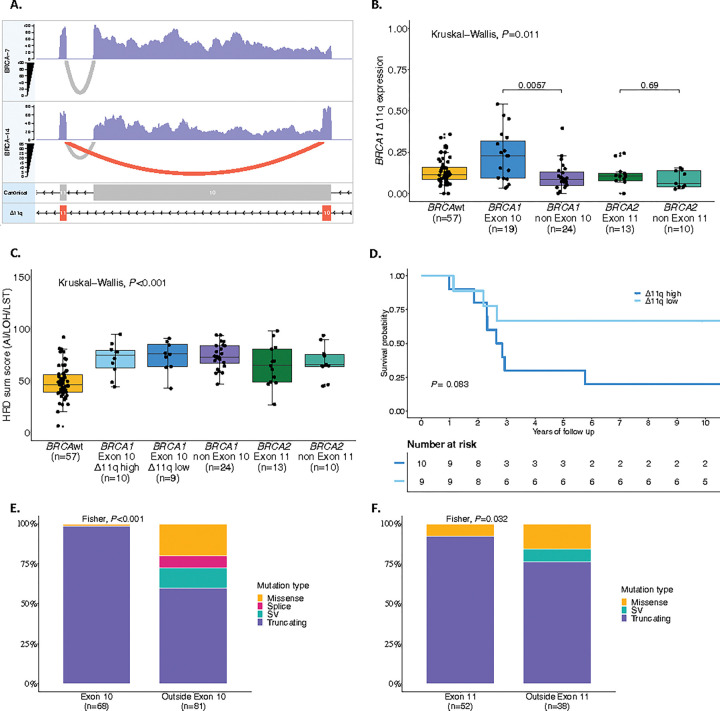
Analysis of pathogenic germline *BRCA1*and *BRCA2* variants and isoform expression on survival in HGSC. **a**, Illustrates the RNA-seq coverage and splice junction reads across the *BRCA1* gene for two samples (BRCA-7 and BRCA-14). The top and middle panels show the expression levels, with BRCA-7 and BRCA-14 indicating overall expression coverage. The bottom panel depicts the structure of the *BRCA1* isoforms, where the canonical isoform includes exon 10, while the Δ11q isoform excludes it. Grey arcs in the top and middle panels represent splice junction reads supporting the canonical isoform, while red arcs indicate reads supporting the Δ11q isoform. The higher expression of the Δ11q isoform in BRCA-14 compared to BRCA-7 highlights differential splicing events between these samples. **b**, Illustrates a comparison of *BRCA1* Δ11q expression among patients with mutations in *BRCA1*exon 10 and outside exon 10, *BRCA2* exon 11 and outside exon 11, and patients with *BRCA* wildtype. Kruskal–Wallis test *P* value is reported as well as pairwise Wilcoxon rank-sum test *P* values. **c**, Shows HRD sum score distribution among patients with mutations in *BRCA1* exon 10 (high and low Δ11q expression) and outside exon 10, *BRCA2* exon 11 and outside exon 11 and *BRCA* wildtype tumors. Kruskal–Wallis test *P* value is reported as well as pairwise Wilcoxon rank-sum test *P* values. **d**, Kaplan-Meier analysis of overall survival comparing high vs low Δ11q expression (divided by median) in patients with a *BRCA1* mutation on Exon 10. *P* value calculated by log-rank test. The distribution of mutation types within *BRCA1* outside exon 10 vs. on exon 10 and for *BRCA2* outside exon 11 vs. on exon 11 is presented in **e** and **f**, respectively. Fisher’s exact test *P* values are reported. BRCAwt=BRCA wildtype, HR=Hazard ratio, n=Number of patients, SV= Structural variants, n= number of patients, LST=Large scale transitions, LOH= Loss of heterozygosity, AI= Allelic imbalance

Patients with *BRCA1* variants in exon 10 have been reported to have poorer outcomes^46^ due to expression of an alternative splice isoform called *BRCA1*-delta11q (Δ11q) that bypasses almost all of exon 10 of *BRCA1* (historically referred to as exon 11). To explore this further, we assessed *BRCA1* isoform expression in our multi-omics cohort (*n* = 154) using the bulk RNA sequencing reads spanning the exon 10 to exon 11 junction (Fig. 2a, Supplementary Tables S7 and S8, Supplementary Information). The Δ11q isoform was widely expressed regardless of *BRCA-*status, but patients with *BRCA1* variants in exon 10 had significantly higher proportions of Δ11q transcripts relative to canonical transcripts (*P* = 0.011; Fig. 2b). Patients with *BRCA1* variants in exon 10 were classified as having high (*n* = 10) or low (n = 9) *BRCA1* Δ11q expression, according to the median. Patients with high Δ11q expression had a shorter survival (median OS 2.74 years) compared to those with low Δ11q expression (median OS not reached), although this was not statistically significant (*P* = 0.083) and was not associated with differences in the HRD sum score (Figs. 2c,d and Supplementary Table S9).

Overall, patients with g*BRCA2*pv had an improved OS compared to non-carriers, regardless of mutation location (Table 3). The only exception was the small group (*n* = 13) with pathogenic variants in the DNA binding domain (DBD) of *BRCA2*, located outside of exon 11, who did not show a statistically significant OS or PFS benefit compared to non-carriers (TR: 0.79 and 0.81, 95% CI: 0.39–1.63 and 0.43–1.51, *P* = 0.528 and *P* = 0.506, respectively).

The type of mutation in *BRCA1* and *BRCA2* also plays a predictive role in response to PARPi therapy in ovarian cancer^43^. In our analysis, pathogenic variants in *BRCA1* exon 10 and *BRCA2* exon 11 were more likely to be truncating (98.6% and 92.3%) than those outside these exons (60% and 76.3%, *P* < 0.001 and *P* = 0.032 respectively; Figs. 2e,f). *BRCA1* and *BRCA2* domains associated with prolonged survival were more likely to have truncating variants than missense or splice site variants (*P* < 0.001 and *P* = 0.067, respectively; Extended Data Figs. 4c,d).

### NF1 gene alterations are associated with improved survival in BRCA2-deficient HGSC

**Figure 3:**
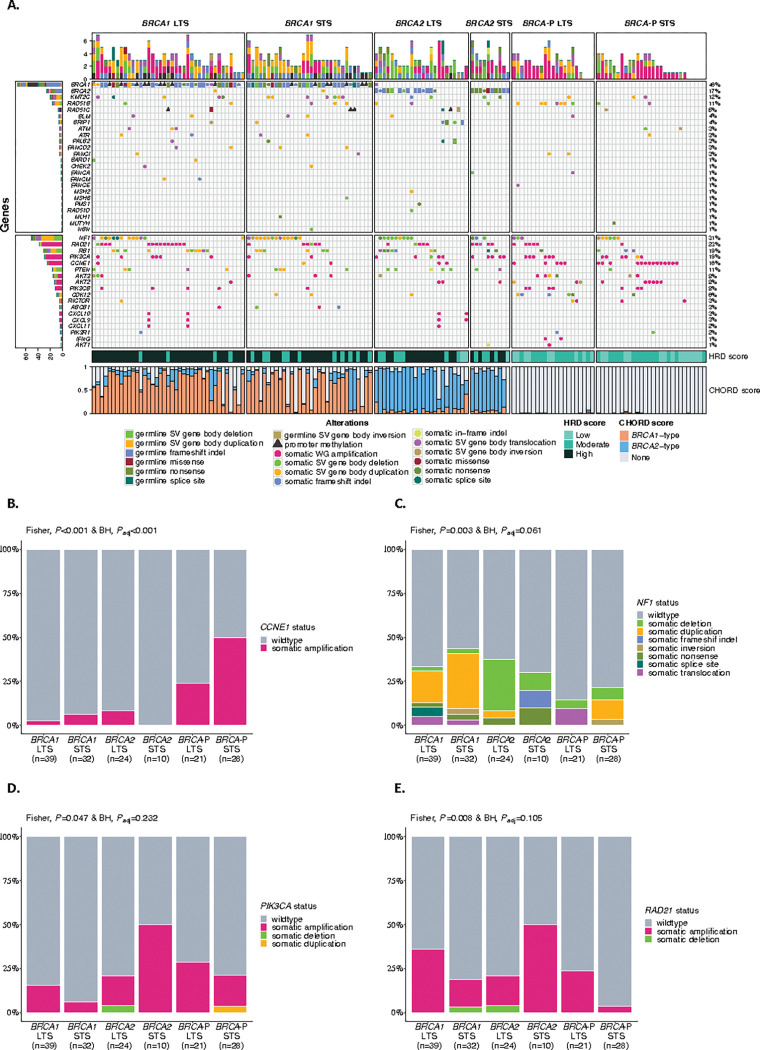
Genetic landscape of HGSC stratified by *BRCA* status and survival. **a**, Oncoprint showing germline and somatic alterations of homologous recombination (HR) genes and other genes of interest stratified by *BRCA*-status and survival group. The distribution of the mutation type within the *BRCA* survival group is shown for **b** *CCNE1*, **c** *NF1*, **d** *PIK3CA*, and **e** *RAD21*. P-values were calculated by the Fisher’s exact test and Benjamini-Hochberg (BH) adjusted (*P*_adj_). BRCA status group: Long-term survivor (LTS) = OS >3 years, Short-term survivor (STS) = OS ≤3 years, BRCA-P=BRCA-proficient, HRD score: High= ≥ 63 HRD Sum, Moderate=42–62, Low= ≤41 HRD Sum, HRD= Homologous recombination deficiency, CHORD= Classifier of HOmologous Recombination Deficiency, SV=Structural variant, WG=Whole gene, BH=Benjamini-Hochberg

To identify genomic features associated with short survival in HRD tumors, we compared tumor genomes and transcriptomes between short (OS ≤ 3 years, STS) and long-term (OS > 3 years, LTS) survival groups (Fig. 3a). Tumor genomes were classified as either *BRCA1*-deficient, *BRCA2*-deficient or *BRCA*-proficient, which incorporated germline and somatic alterations in *BRCA1* and *BRCA2*, as well as other well-defined HR genes, and tumor HRD status as determined by a mutational signature-based classifier (CHORD, Classifier of HOmologous Recombination Deficiency)^60^ (Supplementary Information and Supplementary Tables S10-S12). *CCNE1* amplifications (gene level copy number ≥ 7) were associated with *BRCA*-proficiency, and particularly the short-survival *BRCA-*proficient group (50%, *P*_adj_<0.001; Fig. 3b). *BRCA*-proficient tumors had less genomic scarring and were associated with an older age at diagnosis compared to *BRCA1*-deficient and *BRCA2*-deficient tumors (Extended Data Figs. 5a,b). Gene methylation has been identified as a prognostic factor in HGSC^61^, but no significantly differentially methylated genes with corresponding up- or down-regulated gene expression were observed between STS and LTS groups in *BRCA1*- and *BRCA2*-deficient tumors (Supplementary Table S13 and Supplementary Information).

Alterations in *NF1* were most common in *BRCA*-deficient tumors, regardless of survival group (*BRCA1* STS 43.8%, *BRCA1* LTS 33.3%, *BRCA2* STS 30%, *BRCA2* LTS 37.5%, *BRCA*-P STS 21.4%, *BRCA*-P LTS 14.3%, *P*_adj_=0.061; Fig. 3c and Supplementary Table S14). Notably, gene breakage caused by large-scale deletions was enriched in *BRCA2*-deficient tumors in the LTS group. We hypothesized that not all alteration types equivalently disrupt gene function. Indeed, only 54.2% (26/48) of *NF1* alterations showed a locus-specific loss of heterozygosity (LOH) suggesting a loss-of-function (Supplementary Table S14 and Supplementary Information). Concordantly, *NF1* mRNA expression varied in tumors according to the type of *NF1* alteration and was particularly depleted in those with locus-specific LOH (*P* < 0.0001; Extended Data Fig. 6a). Patients with tumors that harbored loss-of-function *NF1* alterations showed an improved survival compared to non-loss-of-function *NF1* alterations (median OS 11.92 years vs 5.17 years, *P* = 0.032; Extended Data Fig. 6b). In particular, the combination of both *BRCA2*-deficiency and loss-of-function *NF1* alteration (*n* = 11) was associated with the best survival outcome (median OS 16.96 years), almost twice as long as those with *BRCA2*-deficient tumors with no loss-of-function *NF1* alteration (median OS 8.84 years; Extended Data Fig. 6c and Supplementary Table S9).

NF1 protein expression was assessed by IHC in a larger cohort enriched for long-term survivors (*n* = 658; Extended Data Fig. 1). NF1 protein loss was observed in 13.37% (*n* = 88/658) of patients and was associated with improved survival compared to retained NF1 expression (median OS 4.70 vs. 3.58 years, *P* = 0.028; Extended Data Fig. 7a). Although there were few patients with NF1 protein loss and germline *BRCA1* (*n* = 21) or *BRCA2* (*n* = 6) pathogenic variants, NF1 loss was associated with better survival in g*BRCA2*pv-carriers (median OS 8.05 years NF1 loss vs. 5.72 years NF1 retained) but not in g*BRCA1*pv-carriers (median OS 4.74 years NF1 loss vs. 4.69 years NF1 retained; Extended Data Fig. 7b). NF1 loss also was associated with a longer survival among non-carriers (median OS 5.01 years NF1 loss vs. 3.36 years NF1 retained; Extended Data Fig. 7b).

In the independent OTTA cohort with *NF1* mRNA expression and survival data available (n = 5666), low *NF1* expression (lowest quantile) was associated with improved survival compared to high expression (2nd to 5th quantiles) (median OS 4.19 vs. 3.56 years, *P* < 0.0001; Extended Data Figs. 1 and 7c). Consistent with the other cohorts, g*BRCA2*pv-carriers with low *NF1* expression (n = 36) showed an improved survival (median OS 6.42 years *NF1* low vs. 5.66 years *NF1* high), while there was no effect in g*BRCA1*pv-carriers (median OS 5.41 years *NF1* low vs. 5.65 years *NF1* high, Extended Data Fig. 7d).

### PIK3CA and RAD21 amplifications are associated with short survival in BRCA2-deficient HGSC

We found an enrichment of *PIK3CA* and *RAD21* gene amplifications in *BRCA2*-deficient tumors in patients with short compared to long survival (*PIK3CA*: 5/10, 50% vs 4/24, 16.7%, *P*_adj_=0.232 and *RAD21*: 5/10, 50% vs 4/24, 16.7%, *P*_adj_=0.105, respectively; Fig. 3d,e). Co-occurrence of *RAD21* and *PIK3CA* amplification was observed in 8.8% (3/34) patients with *BRCA2*-deficiency (Supplementary Tables S15 and S16). *PIK3CA* and *RAD21* mRNA expression was highly correlated with copy number (*P* < 0.0001), and tumors with gene amplification (≥ 7 copies) had a significantly higher expression (*P* < 0.001 and *P* = 0.02, respectively) (Extended Data Fig. 8a,b and Supplementary Tables S17 and S18). Patients with combined *BRCA2*-deficiency and *PIK3CA* amplification (*n* = 9, median OS 2.89 years) or *RAD21* amplification (*n* = 9, median OS 2.89 years) had a significantly worse prognosis compared to patients with *BRCA2*-deficient tumors without *PIK3CA* amplification (n = 25, median OS 11.92 years) or *RAD21* amplification (*n* = 25, median OS 11.53 years; Extended Data Fig. 8c,d and Supplementary Table S9).

PI-3 kinase pathway activity is thought to contribute to tolerance to genome doubling and *PIK3CA* amplification in whole-genome duplicated tumors is a frequent event in HRD end-stage HGSC^49,62^. The STS *BRCA2*-deficient group was characterized by high ploidy (*P*_adj_=0.0073) and whole-genome duplication (*P*_adj_=0.0404), in contrast to *BRCA1*-deficient and *BRCA*-proficient tumors where the LTS groups tended to have higher ploidy (Extended Data Fig. 5a). The association between *PIK3CA* and survival by *BRCA* status was further corroborated in the OTTA cohort, where g*BRCA2*pv carriers with high *PIK3CA* RNA expression (highest quantile) had shorter survival relative to their counterparts with low expression (median OS 4.09 vs 7.43 years, *P* < 0.0001; Extended Data Fig. 8e). By contrast, g*BRCA1*pv carriers with high *PIK3CA* RNA expression showed improved survival (median OS 7.67 vs 5.23 years).

### Elevated HRD scarring is prognostic for survival in BRCA-deficient HGSC

**Figure 4:**
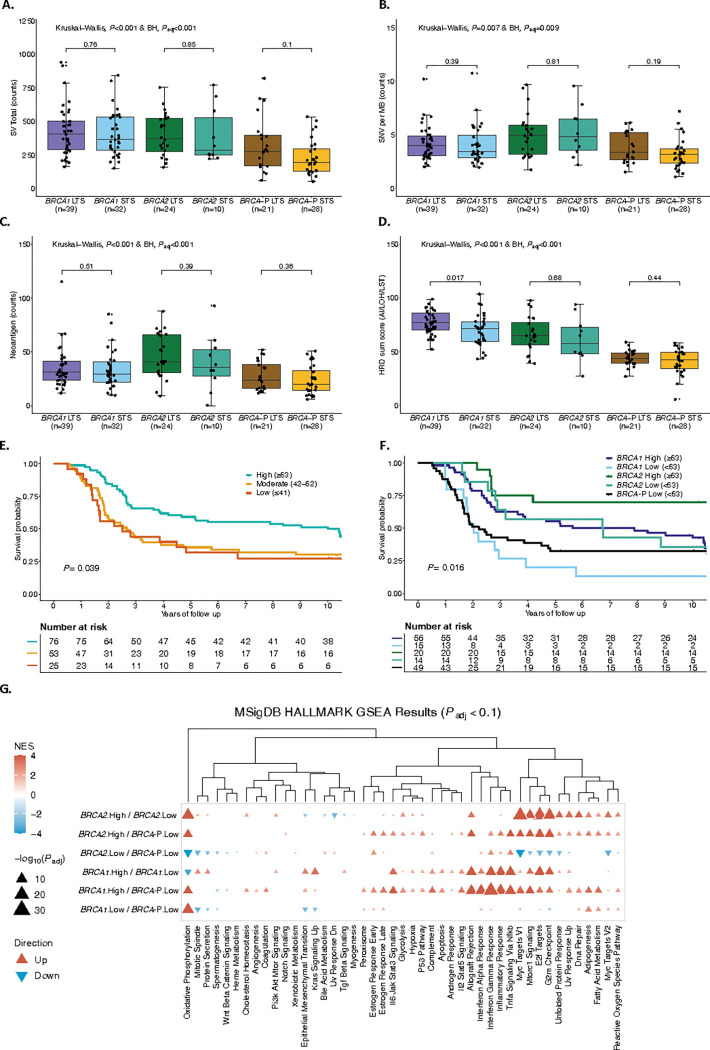
Influence of homologous recombination deficiency in HGSC independent of *BRCA* status. Comparison of **a** SV total counts, **b** SNV counts per megabase, **c** neoantigen counts, and **d** HRD sum score between *BRCA* survival groups (*BRCA1=BRCA1*-deficient; *BRCA2=BRCA2*-deficient; *BRCA-P*=*BRCA*-proficient; Long term survivor (LTS) = OS >3 years; Short term survivor (STS) = OS ≤3 years). *P*-values were calculated by the Kruskal Wallis test and Benjamini-Hochberg (BH) adjusted (*P*_adj_). **e**, Kaplan-Meier analysis of overall survival stratified by different thresholds of the HRD sum score (High ≥63, Moderate 42–62, Low ≤42) in 154 patients with *BRCA*-deficient and *BRCA*-proficient HGSC. *P* value calculated by log-rank test. **f**, Kaplan-Meier analysis of overall survival in patients with HGSC stratified by *BRCA*-status and high (High ≥63) or low (Low < 63) HRD sum score. *P* value calculated by log-rank test. **g**, Clustered heatmap summarizing gene set enrichment analysis (GSEA) using the hallmark Molecular Signatures Database (MSigDB) gene sets. Direction and color of triangles relate to the normalized enrichment score (NES) as generated by FGSEA. P values (two-sided) were calculated using the FGSEA default Monte Carlo method; the size of the triangles corresponds to the negative log10 Benjamini-Hochberg (BH) adjusted P value (*P*_adj_). Columns are separated by *BRCA-*status and HRD score groups (*BRCA1*; *BRCA2*; *BRCA*-P, *BRCA*-proficient, High ≥ 63; Low <63) with the direction of enrichment indicated by the first group mentioned in the x-axis label. SV=Structural variants, SNV=Single nucleotide variant, MB=Megabase, HRD=Homologous recombination deficiency, HRP=Homologous recombination proficiency, BRCA-P=BRCA-proficient, LST=Large scale transitions, LOH= Loss of heterozygosity, AI= Allelic imbalance

High tumor mutation burden has been shown to be associated with long-term survival in ovarian cancer^22^. However, we found that tumor mutation burden and predicted neoantigen counts were equivalent in *BRCA1*-deficient and *BRCA2*-deficient tumors between STS and LTS groups (Fig. 4a-c, Extended Data Fig. 5a, and Supplementary Table S19). Among various genomic features that were compared between these groups (Extended Data Fig. 5a), the HRD score^27^ was elevated in *BRCA1*-deficient tumors with long survival times compared to those with short survival times (*P* = 0.017; Fig. 4d). HRD score is a measure of genomic scarring associated with impaired HR repair, suggesting a more profound inactivation of the HR pathway in patients with good outcome. Retention of the wildtype allele with absence of locus specific LOH has been reported to influence outcomes in g*BRCA*pv-carriers in ovarian and breast cancer^63–66^. However, in our cohort there was only one g*BRCA2*pv carrier without loss of the wildtype allele (patient BRCA_9; Supplementary Table S11 and Supplementary Information). Concordantly this tumor was HR-proficient with an HRD score of 27 (HRP ≤ 42 HRD sum score) and CHORD score of 0 (HRP ≤ 0.5 CHORD score), and the patient had short OS (< 3 years).

We observed a dynamic range in HRD scores, even among tumors with pathogenic *BRCA* mutations, suggesting a non-equivalence of alterations. The cutoff of the HRD score has been debated, with 42 mainly used in recent clinical trials^67–71^, and a more stringent threshold of 63 has been proposed for ovarian cancer^72^. Indeed, patients whose tumors had a high HRD score (≥ 63) had longer OS (median OS 10 years) compared to those with HRD scores of 42–62 (median OS 2.66 years) and ≤ 41 (median OS 2.5 years), regardless of *BRCA*-status (*P* = 0.039; Fig. 4e and Supplementary Table S9). Applying a threshold of 63 to divide samples into high and low HRD, all *BRCA*-proficient tumors had a low HRD score. Furthermore, patients with *BRCA1*- and *BRCA2*-deficient tumors and HRD scores ≥ 63 had longer OS compared to patients with lower HRD scores (median OS 6.76 vs. 2.01 years and 11.88 vs. 6.73 years, respectively; Fig. 4f and Supplementary Table S9). Notably, patients with *BRCA1*-deficient tumors with HRD scores < 63 had similar OS to patients with *BRCA*-proficient tumors (median OS 2.01 years vs 2.21 years).

Gene set enrichment analysis^73^ (GSEA; Methods) revealed distinct patterns of pathway regulation based on HRD scores and *BRCA* status in patients with HGSC. Specifically, pathway activation in *BRCA1*- and *BRCA2*-deficient patients with low HRD (< 63) closely resembled those of *BRCA*-proficient patients (Fig. 4g). In contrast, *BRCA1*-deficient patients with high (≥ 63) HRD scores showed an upregulation of several pathways, including interferon-gamma and inflammatory response. These pathways are primarily involved in host defense and immune surveillance^74^, underscoring their potential role in modulating the tumor microenvironment and influencing immune response in patients with *BRCA1*-deficient tumors.

### CD8 + PD-1 + T cells are prognostic for survival in gBRCApv-carriers

We considered whether *BRCA*-deficient cases with shorter survival would have fewer mutation-associated neoantigens to drive anti-tumor responses, but there was no difference in neoantigen counts between the STS and LTS groups for both *BRCA1* and *BRCA2* (*P* = 0.51 and *P* = 0.39, respectively; Fig. 4c). Tumor samples from 143 HGSC g*BRCA*pv-carriers were analyzed by multi-color immunofluorescence to determine the epithelial and stromal immune cell densities and their associations with survival groups (Extended Data Fig. 1). Aside from intraepithelial B cells and CD4 + T cells (OR = 1.0), all other immune cell subsets had a positive association with survival (OR < 1.0; Supplementary Table S20). Only intrastromal and intraepithelial CD8 + PD-1 + T cells were significantly more abundant in g*BRCA*pv-carriers with LTS compared to those with STS (*P* = 0.043 and *P* = 0.029, respectively; Supplementary Table S20).

### The mesenchymal features c-KIT and mast cells are associated with poor outcome in HGSC

**Figure 5:**
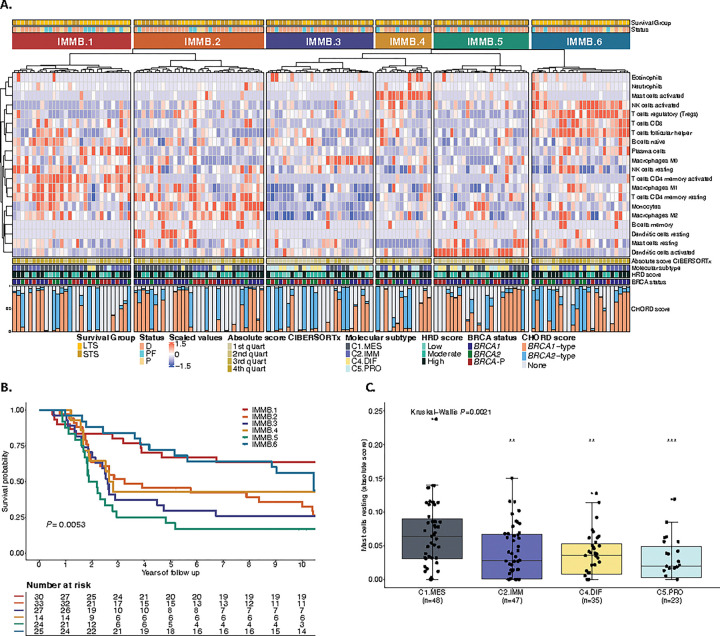
Integration of immune cell profiling by CIBERSORTx and survival analysis in HGSC. **a**, Summary of the immune cell types arising from the CIBERSORTxanalysis from *BRCA-*deficient and *BRCA*-proficient samples (n = 153 patients). Tumors fell into 6 major clusters (IMM.1-IMM.6) of immune cell types associated with survival. Each patient is annotated with survival group, status at last follow-up, CIBERSORTx absolute immune scores, molecular subtype, HRD score, *BRCA* status and CHORD score. **b**, Kaplan-Meier analysis of overall survival stratified by immune clusters. *P*value calculated by log-rank test. **c**, Boxplots summarize the absolute cell enrichment score of mast cells resting markers across the molecular subtype (C1.MES; C2.IMM; C4.DIF; C5.PRO); points represent each sample, boxes show the interquartile range (25–75th percentiles), central lines indicate the median, and whiskers show the smallest/largest values within 1.5 times the interquartile range. Kruskal–Wallis test *P* value is reported as well as pairwise Wilcoxon rank-sum test *P*values comparing molecular subtypes (C2.IMM; C4.DIF; C5.PRO) to C1.MES (**, *P*<0.01, ***, *P* <0.001). Survival group: Long term survivor (LTS)= OS >3 years, Short term survivor (STS)= OS ≤3 years, HRD=Homologous recombination deficiency, HRD score: High= ≥ 63 HRD Sum, Moderate=42–62, Low= ≤41 HRD Sum, Molecular subtypes: C1.MES=C1 mesenchymal subtype, C2.IMM=C2 immunoreactive subtype, C4.DIF=C4 differentiated subtype, C5.PRO=C5 proliferative subtype, Status: D=Dead, PF=Progression-free, P=Progression, IMMB=Immune cluster BadBRCA (IMMB.1-IMMB.6),

Immune cell abundance was estimated in 154 HGSC tumor samples using CIBERSORTx^75^. Unsupervised clustering of the inferred immune cell densities identified six groups of patients (Fig. 5a, and Supplementary Table S21) associated with differential survival outcomes (*P* = 0.0053; Fig. 5b). The IMMB.1 (*n* = 30) and IMMB.6 (*n* = 25) clusters had exceptionally long survival (median OS 14.87 and 10.45 years, respectively; Supplementary Table S9). The group with the shortest survival (cluster IMMB.5, *n* = 24, median OS 2.03 years) was enriched with activated dendritic cells and resting mast cells, a feature associated with the C1.MES subtype (*P* = 0.0021; Fig. 5c). Multivariable Cox regression analysis showed that resting mast cells (HR: 1.26, 95% CI 1.06–1.5, *P* = 0.009) were the immune cell type most strongly associated with short survival (Extended Data Fig. 9a). *BRCA1*-deficient tumors in patients with STS had increased expression of the mast cell growth factor receptor *c-KIT* (CD117) compared to those with LTS (*P* = 0.003, *P*_adj_=0.101; Extended Data Fig. 9b). Patients with high *c-KIT* tumor expression had significantly shorter OS than those with low *c-KIT* tumor expression, regardless of *BRCA* and HRD status (HR: 1.71, 95% CI 1.16–2.53, *P* = 0.0071; Extended Data Fig. 9c). The C1.MES subtype showed higher expression of *c-KIT*, together with an upregulation of the epithelial mesenchymal transition (EMT) pathway, compared to the C2.IMM subtype (*P*_adj_<0.001) (Extended Data Fig. 9d,e).

## DISCUSSION

Our study highlights the complexity of survival determinants in patients with HGSC, demonstrating that it is the intersection of multiple factors, including surgical residual disease, immune response, and somatic gene alterations, which may influence outcome rather than *BRCA* mutation status alone. This interplay was particularly apparent in the diminished adverse impact of surgical residual disease in g*BRCA*pv-carriers compared to non-carriers. Previous reports have suggested that surgery in a *BRCA*-deficient setting may have a lesser impact on survival in both first-line and platinum-sensitive setting^33,47,76^, indicating that it may be particularly important to achieve complete resection of *BRCA*-proficient tumors. In addition, an exploratory analysis of the PAOLA-1/ENGOT-ov25 trial^77^ showed that patients with *BRCA*-proficient tumors classified as higher risk (FIGO stage III with primary cytoreductive surgery and residual disease, or NACT; FIGO stage IV) had notably worse PFS compared to lower-risk patients, while this difference was less pronounced in patients with *BRCA*-deficient tumors. These results emphasize the importance of primary cytoreductive surgery with complete resection for non-carriers, who may also benefit more from secondary cytoreductive surgery in contrast to g*BRCA*pv-carriers^78^. Equally, it may be that the positive effect of optimal cytoreduction is not as apparent in *BRCA* carriers, due to the chemotherapy (platinum) sensitivity associated with *BRCA*-deficiency.

In the current study, the association between NACT and survival appeared to differ by g*BRCA*pv status, with a potential attenuation of survival benefit among g*BRCA*pv-carriers who received NACT. However, the subgroup analyses by g*BRCA*pv status and treatment type were likely underpowered, limiting definitive conclusions regarding potential interactions. Given the rapid increase in the uptake of NACT in recent years^79^, it will be important to determine if patients with *BRCA*-deficient tumors may be negatively impacted by NACT^80^. The acquisition of *BRCA* reversion mutations is frequent^48–50^, and it is plausible that reversion events may be more common where chemotherapy commences with a large tumor volume from which resistant clones could emerge under selection^58^. This is especially important in the PARPi era, where the early development of platinum resistance could negatively impact on the potential benefit gained from PARPi treatment. While the impact of NACT on outcomes according to *BRCA* status is not yet known, it is becoming increasingly important to more rapidly determine the *BRCA* and broader HR status of a patient’s tumor at diagnosis to make the most informed decisions at primary treatment.

Our study highlights the spectrum of HRD scores seen in patients with *BRCA*-deficient tumors. While all but two exceeded a threshold (> 42) required for classification as HRD, the improved OS and PFS seen with a more stringent threshold (≥ 63) shows that HRD should not be considered a binary classification but rather appears to be a continuous variable. This finding is consistent with a previous analysis of 537 HGSC cases from The Cancer Genome Atlas which showed that patients with HRD scores ≥ 63 were associated with better survival outcomes, while those with intermediate (42–62) and low (≤ 42) HRD scores had overlapping survival curves^72^. It is important to mention that in our study, samples were collected over nearly 20 years, a timeframe that encompasses changes in treatment practices, making it challenging to determine how evolving therapies, particularly the introduction of PARPi, may have influenced outcomes. It is notable that the HRD score threshold of 42 was originally established to predict response to neoadjuvant platinum-containing chemotherapy in patients with breast cancer^81^, which tends to have less genomic scarring compared to ovarian cancer^27,72^. As HRD scores ≥ 63 strongly predicted better outcomes in *BRCA*-deficient HGSC, our findings support the prognostic value of HRD score thresholds. However, it is premature to conclude that a higher threshold should alter therapy selection. To establish this, a comprehensive analysis of maintenance PARPi trials, incorporating HRD scores, would be necessary to confirm their predictive role in guiding treatment decisions. Furthermore, it would be ideal to extend this investigation to include other relevant genomic alterations identified in trial samples to refine patient stratification further. This refinement would help identify patients for whom no maintenance therapy or additional targeted therapy may be more appropriate, while avoiding potentially ineffective treatments for those with lower HRD scores, thereby personalizing therapy to maximize efficacy and minimize unnecessary side effects.

Our analyses corroborated Labidi-Galy et al.’s findings that pathogenic variants in the RAD51-BD of *BRCA2*^44^ and the DBD of *BRCA1*^43^ are associated with improved outcomes in HGSC. By contrast, alterations outside *BRCA1* exon 10, particularly in the BRCT and RING regions, are not associated with a significantly improved survival compared to non-carriers and in some cases may confer platinum and PARPi resistance^45^. While *BRCA1* exon 10 mutations have been associated with improved outcomes in multiple studies, including ours, there is evidence that tumors may express the *BRCA1*-Δ11q splice isoform, which bypasses exon 10 mutations and results in a shorter but partially functional protein that is permissive of treatment resistance^43,46^. In a relatively small sample size for which we had RNA-seq data (n = 19 *BRCA1* exon 10 mutated tumors), we found that patients with a pathogenic *BRCA1* variant in exon 10 and high Δ11q expression had a shorter survival. We were unable to measure Δ11q expression during or following treatment. This is important because Δ11q expression may increase or fluctuate under the selective pressure of treatment, which would influence treatment response and survival outcomes.

CD8 + PD1 + T cells are associated with improved outcomes in ovarian cancer^82^, contributing to enhanced anti-tumor immunity. In our analysis, the presence of these cells in tumors were prognostic for survival in g*BRCA*pv-carriers, although to a lesser extent. This suggests that while cytotoxic T-cell activity remains important in *BRCA*-deficient tumors, additional factors may influence survival. Given the established association between *BRCA* and HR status and increased TMB^22^, it is possible that immune exhaustion, suppressive signaling or tumor-intrinsic immune resistance pathways may counteract the expected immunogenicity. Intriguingly, *BRCA1*-deficient tumors with high HRD scores had evidence of enhanced immune-related gene transcription. In addition, while our study did not include cigarette smoking in the survival models, smoking has been identified as a potential factor influencing survival in g*BRCA*pv-carriers^83^, which may also influence the immune response. Further research into markers of T-cell exhaustion and other immune regulators is needed to better understand the differential immune responses in these patients.

NF1 gene loss-of-function emerged as a good prognostic factor in *BRCA2*-deficient HGSC. Loss-of-function of *NF1* is common in epithelial ovarian cancer with a prevalence of 12–31%^13,20,22,58,84,85^. NF1 inactivation by gene breakage or mutations may contribute to initial good prognosis but later chemoresistance in patients with HGSC and *BRCA*-deficiency^84^. This is consistent with recent findings that deleterious *NF1* mutations are associated with improved PFS in ovarian cancer^20^ and low mRNA expression of *NF1* predicts longer overall survival^22^. In contrast, *PIK3CA* amplification and high mRNA expression were associated with shorter survival in patients with *BRCA2*-deficient HGSC. As a major regulator of the phosphoinositide 3-kinase (PI3K) pathway, *PIK3CA* activation promotes cell proliferation and survival, especially in genomically unstable cancers^49,62^. Its amplification may enhance tolerance to genome doubling and contribute to the aggressive nature of *BRCA2*-deficient tumors. The contrasting survival outcomes between *PIK3CA* amplification and *NF1* loss-of-function underscore the heterogeneity of HGSC tumors, highlighting the need for personalized therapeutic strategies, even within the *BRCA2*-deficient subgroup.

## METHODS

### Ethics statement

Written informed consent or an approved waiver of consent was obtained at each participating study site for patient recruitment and the use of samples and linked clinical information (Supplementary Table S22). Investigations were performed after approval by local human research ethics/institutional review board committees at each site. This study was conducted in accordance with the principles of Good Clinical Practice, the Declaration of Helsinki and local regulations.

### Study population

This retrospective, multi-center study included patients diagnosed with HGSC between 2002 and 2019. The Australian Ovarian Cancer Study (AOCS) cohort (n = 1389) included all stages (FIGO I-IV), and the Multidisciplinary Ovarian Cancer Outcomes Group (MOCOG) cohort (n = 154) was restricted to advanced stage disease (FIGO III and IV; Table 1, Extended Data Fig. 1, and Supplementary Table S22). Patients were categorized based on OS into short (< 3 years) and long (≥ 3 years) OS groups (Supplementary Information). For multi-omics analysis, 154 patients had fresh-frozen tumor obtained during primary cytoreductive surgery and matched blood samples, or were previously analyzed^22,58^. Findings were validated in an independent HGSC cohort (n = 5875) from the Ovarian Tumor Tissue Analysis Consortium (OTTA) for which g*BRCA*pv status was available.

### Molecular data

#### Single-nucleotide polymorphism (SNP) arrays

Tumor and matched normal DNA was analyzed with the Infinium OmniExpress-24 BeadChip arrays as described previously^22^. The concordance of normal and tumor DNA was assessed using HYSYS^86^. Tumor DNA samples with estimated tumor cellularity > 40% (determined by qPure^87^ and ASCAT^88^) were considered appropriate for whole genome sequencing and methylation arrays.

##### Whole genome sequencing (WGS)

For WGS, libraries were generated from tumor and matched normal genomic DNA from peripheral blood mononuclear cells with a minimum base coverage of 60x and 30x, respectively. FASTQ files were assessed for sequencing quality using FASTQC (v0.11.8) and, for contaminants using FastQ Screen^89^ (v0.11.4). Adapters, N-content and low-quality bases were trimmed using fastq-mcf (v1.05). Sequenced data was mapped to the human genome reference GRCh37 b37 using the aligner BWA mem^90^ (v.0.7.17-r1188). Aligned BAM files per lane were then sorted, merged and duplicates marked using Picard Tools (v.2.17.3). Further processing of the aligned files included base recalibration using GATK BaseRecalibrator (v4.0.10.1). Coverage calculation was performed using GATK DepthOfCoverage (v3.8–1-0-gf15c1c3ef). GATK HaplotypeCaller (v.4.0.10.1) was used on germline BAMs to generate Genomic Variant Call Format (GVCF) files which were used as the Panel of Normals (PoN) in the Mutect2 somatic variant calling workflow. Tumor purity and ploidy were estimated using FACETS^91^.

#### RNA-sequencing (RNA-seq)

Extracted RNA from tumor tissue samples underwent RNA-seq, with initial quality control checks on raw FASTQ files performed using FastQC^89^ (v0.11.8). Adapter, poly (A) tails, N content and low quality base trimming was done using fastq-mcf (v1.05), and contamination was assessed using FastQ Screen^89^ (v(0.11.4). Reads were then mapped to the human reference GRCh37.92 using the STAR^92^ (v2.6.0b) two-pass method. The mapped reads were then sorted using Picard Tools (v2.17.3). Counts were generated using HTSeq^93^ (v0.10.0) on the GRCh37.92 Ensembl release gene annotation. Raw count data was then subsetted to protein coding genes and lowly expressed genes were removed using the following strategy. First, raw counts were converted to CPM (counts per million) and only protein coding genes with a CPM of greater than 0.5 in at least 10 samples were retained. The resulting raw count matrix was then normalized using the trimmed mean of M values (TMM) method using edgeR^94^ (v3.28.1). Batch effects were removed using limma’s^95^ (v3.48.2) removeBatchEffect function. Batch effect removal was done by applying batch correction on the library type (stranded/unstranded) while preserving the survival group (long/short).

#### Methylation arrays

The generation and processing of methylation array data was performed as previously described by Garsed et al.^22^. Briefly, initial quality control was performed by QuantiFluor (Promega). Subsequently, 500 ng tumor DNA was converted using the EZ DNA Methylation kit (Zymo Research) and analyzed using the Infinium MethylationEPIC BeadChip arrays. The R package minfi^96^ (v1.32.0) was then used for quality control assessment and processing of the methylation data as previously described^22^.

#### Immunofluorescence (IF) data

Tissue microarrays (TMAs) were constructed from formalin-fixed paraffin-embedded (FFPE) blocks of tumor tissue and stained by IF with two panels of antibodies against immune markers of interest. Panel 1 detected CD3, CD8, CD20, FOXP3 and CD79; panel 2 detected CD3, CD8, PD-1, PD-L1 and CD68. Both panels also detected pan-cytokeratin to identify tumor epithelium. Automated cell scoring, including separation of epithelial and stromal regions, was performed using QuPath (v0.2m2), with extensive manual training and validation. CD4 + T cells were defined as CD3 + CD8− cells, as previously^97^.

##### *Immunohistochemistry (IHC)*:

Sections of 4 μm thickness were cut from previously constructed TMAs of FFPE tumor samples. Deparaffinized sections were stained with the C-terminal NF1 antibody (clone NFC, SIGMA #MABE1820; St. Louis, MO, USA) using our previously described protocol on a DAKO Omnis platform: 30 min of pre-treatment heat-induced antigen retrieval in Tris-EDTA buffer, pH = 9.0; primary antibody incubation for 1h at dilution 1/50, 10 min of a mouse linker, and 30 min for the peroxidase labelled Dako EnVision + polymer-based detection system (Dako protocol 1 h-10M-30, Agilent, Santa Clara, CA, USA)^85^. Samples were scored as follows: inactivated (loss of expression with retained internal control), normal retained expression, subclonal loss, uninterpretable (loss of tumor expression but no internal control present), and exclude (no tumor in core) (**Supplementary Information**).

#### mRNA expression data by NanoString

Tumor mRNA expression data for genes of interest (*NF1, PIK3CA, c-KIT*, and *RB1*) and transcriptional molecular subtypes in the OTTA cohort were determined using NanoString, as previously described^98,99^.

### Measurements

#### Variant detection and annotation

Variant calling was performed for:

1. germline base substitution and INDEL variants by VarDictJava^100^ (v1.5.7 with –r = 2 –Q = 10 –f = 0.1).
2. somatic base substitution and INDEL variants using four separate variant callers as follows: by Mutect2^101^ (v4.0.11.0 with defaults), VarDictJava^100^ (v1.5.7 with –r = 2 –Q = 10 –V = 0.05 –f = 0.01), Strelka2^102^ (v2.9.9 with defaults), and VarScan2^103^ (SAMtools^104^) v1.9 for mpileup and VarScan2 v2.4.3 with -min-coverage 7 -min-var-freq = 0.05 -min-freq-for-hom = 0.75 -p-value = 0.99 -somatic-p-value = 0.05 -strand-filter = 0). Variant calls were decomposed and normalized using vt^105^ GATKs ReadBackPhasing tool (v3.8–1-0-gf15c1c3ef with -phaseQualityThresh = 10 – enableMergePhasedSegregatingPolymorphismsToMNP -min_base_quality_score = 10 -min_mapping_quality_score = 10 -maxGenomicDistanceForMNP = 2) was applied on the passing variants per tool to combine contiguous SNVs to MNVs (multi-nucleotide variants). GATK’s CombineVariants (v3.8–1-0-gf15c1c3ef with -genotypeMergeOptions UNIQUIFY -priority Strelka2, Mutect2, VarScan2, VarDictJava) was used to merge the variant calls from all four callers into a consensus variant call set. The resulting variant call format (VCF) file was once again decomposed and normalized using vt. Forward and reverse strand counts for the reference and alternate alleles were calculated using bam-readcount (v0.8.0). Finally, all variants were annotated for Duke and DAC blacklisted regions. Any variants that were passed in at least two callers, had at least one variant read in each strand, and were not in the database of FrequentLy mutAted GeneS (FLAGS)^106^ or the Duke and DAC blacklist regions were deemed high-confidence.
3. structural variants (SV) using four separate callers Manta^107^ + BreakPointInspector (v1.5.0), GRIDSS^108^ (v2.0.1), Smoove (v0.2.2) and SvABA^109^ (v134). The SV calls were split into germline and somatic VCFs per caller. The findBreakpointsOverlaps method of the R library StructuralVariantAnnotation (v1.3.1) with a value of 10 for the ‘maxgap’ parameter was used to intersect common breakpoints between the callers. SVs were annotated to constituent types (duplication, deletion, inversion or translocation) using a simple annotation script provided by the GRIDSS tool. High-confidence SVs were categorized as those called by two or more callers. 4. copy number variations (CNV) detection by FACETS^91^ and cnv_facets (v0.13.0) as described previously^22^. The detected variants were filtered for variants with a high probability of pathogenicity as described in detail before^22^.

#### Mutation burden and downsampling

We downsampled the higher coverage tumor BAM files using Picard DownsampleSam (v2.17.3) to achieve balanced median coverage sequencing batches, to compare mutation burden across samples with inconsistent coverage^22^. The median coverage of the International Cancer Genome Consortium (ICGC) tumors was 52.15x, the MOCOG tumors was 77.81x and the short survival *BRCA* dataset tumors was 64.98x. So, to get the same median coverage across the three batches we downsampled the MOCOG and short survival *BRCA* dataset tumors to the ICGC median by specifying downsampling fractions of 0.67 and 0.8 respectively. See **Supplementary Table S19** for details on the tumor sample coverage before and after downsampling and the number of SNVs, MNVs, indels and SVs called after downsampling.

#### Neoantigen prediction

Neoantigen prediction was performed as previously reported by Garsed et al.^22^. Briefly, HLA-VBSeq^110^ (v11_22_2018) was used to generate HLA types which were then used to identify and construct neoantigen using pVACtools^111^ pVACseq (v1.3.5).

#### Homologous recombination deficiency (HRD)

HRD status was determined using 1) scarHRD^112^, which uses loss of heterozygosity (LOH), telomeric allelic imbalance (TAI), and large scale state transition (LST) in tumor genomes to generate a HRD sum score, and 2) CHORD (Classifier of Homologous Recombination Deficiency)^60^, which uses specific base substitution, indel and structural rearrangement signatures detected in tumor genomes to generate *BRCA1*-type and *BRCA2*-type HRD scores.

#### RNA-seq data analysis

Raw count data was subsetted to protein coding genes and lowly expressed genes were removed using the following strategy. First, raw counts were converted to CPM (counts per million) and only protein coding genes with a CPM of greater than 0.5 in at least 10 samples were retained. The resulting raw count matrix was then normalized using the trimmed mean of M values (TMM) method using edgeR^94^ (v3.28.1). Batch effects were removed using limma’s^95^ (v3.48.2) removeBatchEffect function. Batch effect removal was done by applying batch correction on the library type (stranded/unstranded) while preserving the survival group (long-term/short-term).

#### RNA differential expression and pathway analysis by grouping

##### Groupings

For differential expression and pathway analysis, various groupings were used alone or in combination, namely 1) *BRCA*-deficiency status, 2) HRD groups, survival groups, and 3) molecular subtypes (**Supplementary Information**).

##### Differential expression analysis

To identify differentially expressed protein-coding genes between the comparison groups of interest, DESeq2 (v1.26.0)^113^ was applied. Raw counts were filtered to remove low expressed genes prior to analysis and batch effects were accounted for in the model^22^.

##### Gene Set Enrichment Analysis (GSEA)

FGSEA v1.15.1 was used to calculate gene set enrichment across the comparison groups. *P*-values obtained from DESeq2 were transformed to signed *P*-values and then sorted and fed into FGSEA to generate enrichment scores and FDR-adjusted *P*-values across the Hallmark gene sets in the MSigDB database49 (v7.4) via its function fgseaMultilevel (minSize = 15, maxSize = 500, gseaParam = 0, eps = 0)^22^.

#### CIBERSORTx

CIBERSORTx analysis was performed as previously described^22^. Briefly, CIBERSORTx^75^ with the LM22 matrix was used on RNA-seq data for immune cell deconvolution. Immune clusters were then generated with k-means clustering of the generated absolute cell abundances using ConsensusClusterPlus^114^ (Supplementary Information).

#### Immunofluorescence

Data were categorized based on epithelial content, measured directly by pan-cytokeratin positivity and cell morphology (assessed by automated image analysis). Epithelium-negative, cellular (i.e., non-necrotic) tumor regions were defined as stroma. Immunomarker density (D; cells/mm^2^) for a given marker was calculated separately for epithelial and stromal compartments. For cases with multiple cores, the epithelial area was taken as the sum of all their individual TMA epithelial areas and similarly for the stromal area. We categorized marker D values into quartiles (separately for epithelial and stromal markers) to provide categorical comparisons for ease of interpretation of the odds ratios (ORs). Conditional logistic regression models were fitted for the long survival group vs short survival group. Logistic regression analyses were performed with the quartile values (scored as 1, 2, 3, 4). Immune clusters were then generated by k-means clustering of the immune cell type densities using ConsensusClusterPlus^114^.

#### Statistical analyses

Continuous variables were compared between groups using the Kruskal-Wallis test and the difference between proportions of categorical data were assessed using the Chi-squared or Fisher’s exact test. Correlations between continuous variables were assessed using Spearman correlation. Benjamini-Hochberg adjusted *P*-values are reported as *P*_adj_ to account for multiple testing. Median PFS and OS were estimated using the Kaplan-Meier method and survival distribution were compared using the log-rank (Mantel-Cox) test.

For the AOCS cohort, univariable and multivariable survival analyses were performed using Accelerated Failure Time (AFT) models^54^ with a log-logistic distribution to evaluate associations between clinical and molecular variables and time-to-event outcomes. Results were reported as Time Ratios (TR) with 95% confidence intervals (CI), where a TR > 1 indicates longer time to progression or death, and a TR < 1 indicates shorter survival. Wald tests were used to compute *P*-values for individual covariates and interaction terms. Age at diagnosis was modelled using restricted cubic splines with three knots to allow for potential non-linear effects. Model assumptions were assessed using quantile-quantile plots of deviance residuals and Cox-Snell residuals to evaluate overall model fit. The Akaike Information Criterion (AIC) was used to compare alternative parametric distributions and confirm the suitability of the log-logistic model^115^.

For survival analyses of the OTTA cohort, Cox proportional hazards models were applied. Left truncation was used to account for delayed study enrolment at some sites, and follow-up time was right-censored at 10 years from diagnosis to minimize the influence of non-ovarian cancer-related deaths. *P*-values from Cox models correspond to Wald and log-rank tests. The proportional hazards assumption was assessed using the Grambsch-Therneau test based on scaled Schoenfeld residuals and further evaluated through graphical inspection of Schoenfeld residual plots^115,116^.

All statistical tests were two sided and considered significant when *P* < 0.05 or *P*_*adj*_ <0.1. All analyses were performed using the statistical software R version 4.1.3^117^.

## Supplementary Material

Supplementary Files

This is a list of supplementary files associated with this preprint. Click to download.

• ExtendedDataFigures.pdf

• SupplementaryInformation.docx

• SupplementaryTables.xlsx

## Figures and Tables

**Table 1:** Baseline characteristics of the clinicopathological features from patients with high-grade serous ovarian cancer (HGSC) of the Australian Ovarian Cancer Study (AOCS) cohort.

Characteristics	n = 1,389 n (%)
**Age at diagnosis (years)**	
Median	61
Range	24–87
Unknown	7 (0.5)
**Germline BRCA status**	
Wildtype	1,107 (79.7)
g*BRCA1*pv	175 (12.6)
g*BRCA2*pv	107 (7.7)
**Grade**	
G3	1,100 (79.2)
G2	237 (17.1)
Unknown	52 (3.7)
**FIGO stage**	
III-IV	1,193 (85.9)
I-II	134 (9.6)
Unknown	62 (4.5)
**Primary site**	
Ovary	1,008 (72.6)
Peritoneum	215 (15.5)
Fallopian tube	140 (10.1)
Unknown	26 (1.9)
**Surgery**	
Primary cytoreductive surgery	991 (71.3)
Interval cytoreductive surgery	299 (21.5)
Other	70 (5)
Unknown	29 (2.1)
**Residual disease status**	
Residual disease	829 (59.7)
No residual disease	467 (33.6)
Unknown	93 (6.7)
**Neoadjuvant chemotherapy**	
No	1,060 (76.3)
Yes	322 (23.2)
Unknown	7 (0.5)
**PARP inhibitor 1st line**	
No	1,350 (97.2)
Yes	39 (2.8)
**Progression-free survival (months)**	
Median	15
Range	0–285
Unknown	11 (0.8)
**Overall survival (months)**	
Median	38
Range	1–290
Unknown	11 (0.8)
**Status**	
Deceased	984 (70.8)
Alive	393 (28.3)
Unknown	12 (0.9)

**Table 2:** Multivariable Accelerated Failure Time (AFT) model of BRCA and residual disease status and clinicopathological predictive features on overall survival in patients from the Australian Ovarian Cancer Study (AOCS) cohort. The model was fitted using a log-logistic distribution. Results are expressed as Time Ratios (TR) with corresponding 95% confidence intervals (CI) and p-values derived from Wald tests. A TR > 1 indicates a longer survival time, whereas a TR < 1 indicates a shorter survival time. Age at diagnosis was modeled using restricted cubic splines with 3 knots and is presented as two spline terms.

				Univariable				Multivariable		
				95%CI				95%CI		
Feature	Factor	Number	TR	lower	upper	*P*-value	TR	lower	upper	*P*-value
g*BRCA*pv & Residual status	Non carriers & R0	356	-	-	-	-	-	-	-	-
	Non carriers & R	649	0.42	0.37	0.48	< 0.001	0.51	0.44	0.59	< 0.001
	g*BRCA*pv carriers & R0	105	1.31	1.03	1.66	0.028	1.18	0.92	1.50	0.191
	g*BRCA*pv carriers & R	174	0.82	0.68	0.98	0.028	0.87	0.72	1.06	0.162
FIGO stage	I + II	134	-	-	-	-	-	-	-	-
	III + IV	1183	0.34	0.28	0.42	< 0.001	0.59	0.47	0.74	< 0.001
Primary site	Ovary	1002	-	-	-	-	-	-	-	-
	FT	135	1.28	1.04	1.58	0.023	1.10	0.89	1.36	0.364
	Peritoneum	215	0.66	0.57	0.77	< 0.001	0.82	0.71	0.94	0.005
Age at diagnosis	Years Spline 1	1370	0.99	0.98	1.00	0.069	1.00	0.99	1.01	0.669
	Years Spline 2		0.99	0.97	1.00	0.142	0.98	0.97	1.00	0.029
Surgery	Primary CS	980	-	-	-	-	-	-	-	-
	Interval CS	299	0.83	0.72	0.95	0.007	0.89	0.48	1.64	0.703
	Other	69	1.27	0.99	1.63	0.065	1.13	0.84	1.53	0.418
Neoadjuvant CHT	No	1048	-	-	-	-	-	-	-	-
	Yes	322	0.83	0.72	0.95	0.006	0.96	0.53	1.75	0.897
Grade	G2	237	-	-	-	-	-	-	-	-
	G3	1088	1.22	1.05	1.41	0.009	1.07	0.93	1.22	0.347
PARP inhibitor 1st line	No	1338	-	-	-	-	-	-	-	-
	Yes	39	1.40	0.90	2.12	0.119	1.25	0.81	1.92	0.321

R = Residual disease, R0 = No residual disease, G2 = Grade 2, G3 = Grade 3, OS = Overall survival, gBRCApv = pathogenic germline BRCA variant, TR = Time ratio, CI = confidence interval, CHT= chemotherapy, CS = cytoreductive surgery, FT= fallopian tube

**Table 3:** Adjusted Accelerated Failure Time (AFT) model analysis of germline *BRCA* pathogenic variant (g*BRCA*pv) location and progression-free survival and overall survival in patients from the Australian Ovarian Cancer Study (AOCS) cohort. Models were adjusted for FIGO stage, residual disease status, primary tumor site, type of surgery, age at diagnosis (modelled with restricted cubic splines, 3 knots), use of neoadjuvant chemotherapy, tumor grade, and PARP inhibitor use in first-line treatment. AFT models were fitted using a log-logistic distribution. Results are presented as Time Ratios (TR) with 95% confidence intervals (CI) and *P*-values derived from Wald tests. A TR > 1 indicates an association with longer time to progression or death, while a TR < 1 reflects shorter survival. The reference group for all comparisons is non-carriers of g*BRCA*pv.

			Progression-free survival				Overall survival			
				95%CI				95%CI		
Feature	Factor	Number	TR	lower	upper	*P*-value	TR	lower	upper	*P*-value
g*BRCA*pv exon	Non carriers	1096	-	-	-	-	-	-	-	-
	g*BRCA1*pv Exon 10	68	1.49	1.16	1.91	0.002	1.54	1.19	2.00	< 0.001
	g*BRCA1*pv outside Exon 10	81	1.18	0.96	1.46	0.12	1.21	0.97	1.51	0.093
	g*BRCA2*pv Exon 11	52	1.52	1.16	2.00	0.002	1.67	1.26	2.23	< 0.001
	g*BRCA2*pv outside Exon 11	38	1.66	1.15	2.42	0.007	1.90	1.31	2.76	< 0.001
g*BRCA*pv domain	Non carriers	1096	-	-	-	-	-	-	-	-
	g*BRCA1*pv BRCT	17	1.43	0.90	2.26	0.126	1.35	0.83	2.20	0.222
	g*BRCA1*pv DBD	40	1.58	1.15	2.18	0.005	1.60	1.14	2.25	0.006
	g*BRCA1*pv outside domain	54	1.41	1.09	1.81	0.007	1.38	1.06	1.79	0.017
	g*BRCA1*pv RING	27	1.15	0.82	1.61	0.419	1.28	0.87	1.90	0.216
	g*BRCA2*pv DBD	13	0.81	0.43	1.51	0.506	0.79	0.39	1.63	0.528
	g*BRCA2*pv outside domain	35	2.10	1.46	3.00	< 0.001	2.03	1.44	2.87	< 0.001
	g*BRCA2*pv RAD51-BD	39	1.37	1.01	1.85	0.04	1.58	1.14	2.21	0.006

DBD = DNA Binding Domain, RING = Really Interesting New Gene, RAD51-BD = RAD51 Binding Domain, BRCT = BRCA1 C-Terminal

## Data Availability

*Short survival BRCA dataset:* WGS, RNA-seq and SNP array data from short-term survivors generated as part of the current study have been deposited in the European Genome-phenome Archive (EGA) repository (https://ega-archive.org) under accession code EGAS00001008059. WGS and RNA-seq data are available as raw FASTQ files for each sample type (tumor/normal) and SNP array data are available as raw signal intensity files in text format for each sample type (tumor/normal). Access to patient sequence data can be gained for academic use through application to the independent Data Access Committee (DGO@petermac.org). Responses to data requests will be provided within two weeks. Information on how to apply for access is available at the EGA under accession code EGAS00001008059. The raw methylation data sets have been submitted to the Gene Expression Omnibus (GEO; https://www.ncbi.nlm.nih.gov/geo/) under accession code GSE292140 (https://www.ncbi.nlm.nih.gov/geo/query/acc.cgi?acc=GSE292140) with no access restrictions. no access restrictions. *ICGC dataset:* Previously published WGS and RNA-seq data generated as part of the ICGC Ovarian Cancer project^58^ are available from the EGA repository as a single bam file for each sample type (tumor/normal), under the accession code EGAD00001000877 (“https://ega-archive.org/datasets/EGAD00001000877”https://ega-archive.org/datasets/EGAD00001000877). Due to the sensitive nature of these patient datasets, access is subject to approval from the ICGC Data Access Compliance Office (https://docs.icgc.org/download/data-access/), an independent body who authorizes controlled access to ICGC sequencing data. ICGC SNP array and methylation data sets have been deposited into the Gene Expression Omnibus (GEO; https://www.ncbi.nlm.nih.gov/geo/) under accession code GSE65821 (https://www.ncbi.nlm.nih.gov/geo/query/acc.cgi?acc=GSE65821), without access restrictions. ICGC gene count level transcriptomic data has been deposited into the GEO under accession code GSE209964 (https://www.ncbi.nlm.nih.gov/geo/query/acc.cgi?acc=GSE209964).https://docs.icgc.org/download/data-access/), an independent body who authorizes controlled access to ICGC sequencing data. ICGC SNP array and methylation data sets have been deposited into GEOhttps://www.ncbi.nlm.nih.gov/geo/ under accession code GSE65821 (https://www.ncbi.nlm.nih.gov/geo/query/acc.cgi?acc=GSE65821), without access restrictions. ICGC gene count level transcriptomic data has been deposited into the GEO under accession code GSE209964 (https://www.ncbi.nlm.nih.gov/geo/query/acc.cgi?acc=GSE209964). *MOCOG dataset:* WGS, RNA-seq and SNP array data from long-term survivors generated as part of the MOCOG study^22^ have been deposited in the EGA repository under accession code EGAS00001005984. WGS and RNA-seq data are available as raw FASTQ files for each sample type (tumor/normal) and SNP array data are available as raw signal intensity files in text format for each sample type (tumor/normal). Access to patient sequence data can be gained for academic use through application to the independent Data Access Committee (DGO@petermac.org). Responses to data requests will be provided within two weeks. Information on how to apply for access is available at the EGA under accession code EGAS00001005984. The MOCOG cohort raw methylation data sets have been submitted to the GEO under accession code GSE211687 (https://www.ncbi.nlm.nih.gov/geo/query/acc.cgi?acc=GSE211687), with no access restrictions. *OTTA dataset:* Participants of this study did not agree to their data being shared publicly; accordingly, the data used in this research will not be made available. Uniformly processed somatic variant data from the ICGC, MOCOG, and short survival *BRCA* cohorts is deposited in Synapse under accession code syn65463502 and processed expression and methylation data from all cohorts has been submitted into the GEO under accession code GSE292140 (https://www.ncbi.nlm.nih.gov/geo/query/acc.cgi?acc=GSE292140) and GSE292142 (https://www.ncbi.nlm.nih.gov/geo/query/acc.cgi?acc=GSE292142https://www.ncbi.nlm.nih.gov/geo/query/acc.cgi?acc=GSE292142, without access restrictions. All other data are available within the article (and its Supplementary Information files) or from the corresponding authors on request. Population frequencies of genetic variants can be accessed via the Genome Aggregation Database (gnomAD) at https://gnomad.broadinstitute.org/. Supporting evidence for pathogenicity of genomic alterations can be accessed via ClinVar (https://www.ncbi.nlm.nih.gov/clinvar/), BRCA Exchange (https://brcaexchange.org/) and the *TP53* Database (https://tp53.isb-cgc.org/). The Ensembl ranked order of severity of variant consequences is available at: https://m.ensembl.org/info/genome/variation/prediction/predicted_data.html. Mutational signature reference databases can be accessed via COSMIC (https://cancer.sanger.ac.uk/signatures/) and Signal (https://signal.mutationalsignatures.com/). The LM22 signature matrix used for immune cell deconvolution can be downloaded here: https://cibersortx.stanford.edu/. MSigDB hallmark gene sets can be accessed here: https://www.gsea-msigdb.org/gsea/msigdb/. Illumina methylation probes that were filtered out due to poor performance (e.g. cross reactive or non-specific probes) can be found here: https://github.com/sirselim/illumina450k_filtering. Germline polymorphic sites for reference and variant allele read counts used in FACETS analysis can be found at ftp://ftp.ncbi.nih.gov/snp/organisms/human_9606_b151_GRCh37p13/VCF/common_all_20180423.vcf.gz. The GTF used for annotation and RNA-seq counts is available here: ftp://ftp.ensembl.org/pub/grch37/release-92/.

## Code availability

No custom code or software was used in the data analyses and for the figures. All results can be replicated using publicly available tools and software. The tools and versions used are described in the Methods and Supplementary Information.

## References

[1] Hollis R. L. Molecular characteristics and clinical behaviour of epithelial ovarian cancers. Cancer Lett 555, 216057 (2023). 10.1016/j.canlet.2023.216057 36627048

[2] Ahmed A. A. Driver mutations in TP53 are ubiquitous in high grade serous carcinoma of the ovary. J Pathol 221, 49–56 (2010). 10.1002/path.2696 20229506 PMC3262968

[3] Kobel M. p53 and ovarian carcinoma survival: an Ovarian Tumor Tissue Analysis consortium study. J Pathol Clin Res 9, 208–222 (2023). 10.1002/cjp2.311 36948887 PMC10073933

[4] Kobel M. Ovarian carcinoma subtypes are different diseases: implications for biomarker studies. PLoS Med 5, e232 (2008). 10.1371/journal.pmed.0050232 19053170 PMC2592352

[5] Dareng E. O. Polygenic risk modeling for prediction of epithelial ovarian cancer risk. Eur J Hum Genet 30, 349–362 (2022). 10.1038/s41431-021-00987-7 35027648 PMC8904525

[6] Barnes D. R. Large-scale genome-wide association study of 398,238 women unveils seven novel loci associated with high-grade serous epithelial ovarian cancer risk. medRxiv (2024). 10.1101/2024.02.29.24303243 PMC12635163 41266372

[7] Winham S. J. Investigation of Exomic Variants Associated with Overall Survival in Ovarian Cancer. Cancer Epidemiol Biomarkers Prev 25, 446–454 (2016). 10.1158/1055-9965.EPI-15-0240 26747452 PMC4779669

[8] Permuth J. B. Exome genotyping arrays to identify rare and low frequency variants associated with epithelial ovarian cancer risk. Hum Mol Genet 25, 3600–3612 (2016). 10.1093/hmg/ddw196 27378695 PMC5179948

[9] Johnatty S. E. ABCB1 (MDR1) polymorphisms and ovarian cancer progression and survival: a comprehensive analysis from the Ovarian Cancer Association Consortium and The Cancer Genome Atlas. Gynecol Oncol 131, 8–14 (2013). 10.1016/j.ygyno.2013.07.107 23917080 PMC3795832

[10] Charbonneau B. Large-scale evaluation of common variation in regulatory T cell-related genes and ovarian cancer outcome. Cancer Immunol Res 2, 332–340 (2014). 10.1158/2326-6066.CIR-13-0136 24764580 PMC4000890

[11] Goode E. L. A genome-wide association study identifies susceptibility loci for ovarian cancer at 2q31 and 8q24. Nat Genet 42, 874–879 (2010). 10.1038/ng.668 20852632 PMC3020231

[12] Song H. A genome-wide association study identifies a new ovarian cancer susceptibility locus on 9p22.2. Nat Genet 41, 996–1000 (2009). 10.1038/ng.424 19648919 PMC2844110

[13] Colombo N. ESMO-ESGO consensus conference recommendations on ovarian cancer: pathology and molecular biology, early and advanced stages, borderline tumours and recurrent disease. Int J Gynecol Cancer (2019). 10.1136/ijgc-2019-000308 31048403

[14] Bowtell D. D. Rethinking ovarian cancer II: reducing mortality from high-grade serous ovarian cancer. Nat Rev Cancer 15, 668–679 (2015). 10.1038/nrc4019 26493647 PMC4892184

[15] Ledermann J. A. Newly diagnosed and relapsed epithelial ovarian carcinoma: ESMO Clinical Practice Guidelines for diagnosis, treatment and follow-up. Ann Oncol 29, iv259 (2018). 10.1093/annonc/mdy157 30285216

[16] Gonzalez-Martin A. Newly diagnosed and relapsed epithelial ovarian cancer: ESMO Clinical Practice Guideline for diagnosis, treatment and follow-up. Ann Oncol (2023). 10.1016/j.annonc.2023.07.011 37597580

[17] Lord C. J. & Ashworth A. BRCAness revisited. Nat Rev Cancer 16, 110–120 (2016). 10.1038/nrc.2015.21 26775620

[18] Lord C. J. & Ashworth A. PARP inhibitors: Synthetic lethality in the clinic. Science 355, 1152–1158 (2017). 10.1126/science.aam7344 28302823 PMC6175050

[19] Heeke A. L. Prevalence of Homologous Recombination-Related Gene Mutations Across Multiple Cancer Types. JCO Precis Oncol 2018 (2018). 10.1200/PO.17.00286 PMC6139373 30234181

[20] Landen C. N. Influence of Genomic Landscape on Cancer Immunotherapy for Newly Diagnosed Ovarian Cancer: Biomarker Analyses from the IMagyn050 Randomized Clinical Trial. Clin Cancer Res 29, 1698–1707 (2023). 10.1158/1078-0432.CCR-22-2032 36595569 PMC10150250

[21] Lord C. J. & Ashworth A. The DNA damage response and cancer therapy. Nature 481, 287–294 (2012). 10.1038/nature10760 22258607

[22] Garsed D. W. The genomic and immune landscape of long-term survivors of high-grade serous ovarian cancer. Nat Genet 54, 1853–1864 (2022). 10.1038/s41588-022-01230-9 36456881 PMC10478425

[23] Mukhopadhyay A. Development of a functional assay for homologous recombination status in primary cultures of epithelial ovarian tumor and correlation with sensitivity to poly(ADP-ribose) polymerase inhibitors. Clin Cancer Res 16, 2344–2351 (2010). 10.1158/1078-0432.CCR-09-2758 20371688

[24] Miller R. E. ESMO recommendations on predictive biomarker testing for homologous recombination deficiency and PARP inhibitor benefit in ovarian cancer. Ann Oncol 31, 1606–1622 (2020). 10.1016/j.annonc.2020.08.2102 33004253

[25] Stiegeler N. Homologous recombination proficient subtypes of high-grade serous ovarian cancer: treatment options for a poor prognosis group. Frontiers in Oncology 14 (2024). 10.3389/fonc.2024.1387281 PMC11183307 38894867

[26] Nguyen L., J W. M. M., Van Hoeck A. & Cuppen E. Pan-cancer landscape of homologous recombination deficiency. Nat Commun 11, 5584 (2020). 10.1038/s41467-020-19406-4 33149131 PMC7643118

[27] Marquard A. M. Pan-cancer analysis of genomic scar signatures associated with homologous recombination deficiency suggests novel indications for existing cancer drugs. Biomark Res 3, 9 (2015). 10.1186/s40364-015-0033-4 26015868 PMC4443545

[28] Fong P. C. Poly(ADP)-ribose polymerase inhibition: frequent durable responses in BRCA carrier ovarian cancer correlating with platinum-free interval. J Clin Oncol 28, 2512–2519 (2010). 10.1200/JCO.2009.26.9589 20406929

[29] Pennington K. P. Germline and somatic mutations in homologous recombination genes predict platinum response and survival in ovarian, fallopian tube, and peritoneal carcinomas. Clin Cancer Res 20, 764–775 (2014). 10.1158/1078-0432.CCR-13-2287 24240112 PMC3944197

[30] Farmer H. Targeting the DNA repair defect in BRCA mutant cells as a therapeutic strategy. Nature 434, 917–921 (2005). 10.1038/nature03445 15829967

[31] Swisher E. M. Rucaparib in relapsed, platinum-sensitive high-grade ovarian carcinoma (ARIEL2 Part 1): an international, multicentre, open-label, phase 2 trial. Lancet Oncol 18, 75–87 (2017). 10.1016/S1470-2045(16)30559-9 27908594

[32] Banerjee S. Maintenance olaparib for patients with newly diagnosed advanced ovarian cancer and a BRCA mutation (SOLO1/GOG 3004): 5-year follow-up of a randomised, double-blind, placebo-controlled, phase 3 trial. Lancet Oncol 22, 1721–1731 (2021). 10.1016/S1470-2045(21)00531-3 34715071

[33] Alsop K. BRCA mutation frequency and patterns of treatment response in BRCA mutation-positive women with ovarian cancer: a report from the Australian Ovarian Cancer Study Group. J Clin Oncol 30, 2654–2663 (2012). 10.1200/JCO.2011.39.8545 22711857 PMC3413277

[34] Bolton K. L. Association between BRCA1 and BRCA2 mutations and survival in women with invasive epithelial ovarian cancer. JAMA 307, 382–390 (2012). 10.1001/jama.2012.20 22274685 PMC3727895

[35] Candido-dos-Reis F. J. Germline mutation in BRCA1 or BRCA2 and ten-year survival for women diagnosed with epithelial ovarian cancer. Clin Cancer Res 21, 652–657 (2015). 10.1158/1078-0432.CCR-14-2497 25398451 PMC4338615

[36] du Bois A. Role of surgical outcome as prognostic factor in advanced epithelial ovarian cancer: a combined exploratory analysis of 3 prospectively randomized phase 3 multicenter trials: by the Arbeitsgemeinschaft Gynaekologische Onkologie Studiengruppe Ovarialkarzinom (AGO-OVAR) and the Groupe d’Investigateurs Nationaux Pour les Etudes des Cancers de l’Ovaire (GINECO). Cancer 115, 1234–1244 (2009). 10.1002/cncr.24149 19189349

[37] Kotsopoulos J. Impact of germline mutations in cancer-predisposing genes on long-term survival in patients with epithelial ovarian cancer. Br J Cancer 127, 879–885 (2022). 10.1038/s41416-022-01840-4 35710751 PMC9428139

[38] Chase D. M., Mahajan A., Scott D. A., Hawkins N. & Kalilani L. The impact of varying levels of residual disease following cytoreductive surgery on survival outcomes in patients with ovarian cancer: a meta-analysis. BMC Womens Health 24, 179 (2024). 10.1186/s12905-024-02977-5 38491366 PMC10941390

[39] Tothill R. W. Novel molecular subtypes of serous and endometrioid ovarian cancer linked to clinical outcome. Clin Cancer Res 14, 5198–5208 (2008). 10.1158/1078-0432.CCR-08-0196 18698038

[40] Kengsakul M. Factors predicting postoperative morbidity after cytoreductive surgery for ovarian cancer: a systematic review and meta-analysis. J Gynecol Oncol 33, e53 (2022). 10.3802/jgo.2022.33.e53 35712967 PMC9250852

[41] Nielsen J. S. CD20+ tumor-infiltrating lymphocytes have an atypical CD27− memory phenotype and together with CD8+ T cells promote favorable prognosis in ovarian cancer. Clin Cancer Res 18, 3281–3292 (2012). 10.1158/1078-0432.CCR-12-0234 22553348

[42] Hwang W. T., Adams S. F., Tahirovic E., Hagemann I. S. & Coukos G. Prognostic significance of tumor-infiltrating T cells in ovarian cancer: a meta-analysis. Gynecol Oncol 124, 192–198 (2012). 10.1016/j.ygyno.2011.09.039 22040834 PMC3298445

[43] Labidi-Galy S. I. Association of location of BRCA1 and BRCA2 mutations with benefit from olaparib and bevacizumab maintenance in high-grade ovarian cancer: phase III PAOLA-1/ENGOT-ov25 trial subgroup exploratory analysis. Ann Oncol 34, 152–162 (2023). 10.1016/j.annonc.2022.11.003 36564284

[44] Labidi-Galy S. I. Location of Mutation in BRCA2 Gene and Survival in Patients with Ovarian Cancer. Clin Cancer Res 24, 326–333 (2018). 10.1158/1078-0432.CCR-17-2136 29084914

[45] Wang Y. RING domain-deficient BRCA1 promotes PARP inhibitor and platinum resistance. J Clin Invest 126, 3145–3157 (2016). 10.1172/JCI87033 27454289 PMC4966309

[46] Wang Y. The BRCA1-Delta11q Alternative Splice Isoform Bypasses Germline Mutations and Promotes Therapeutic Resistance to PARP Inhibition and Cisplatin. Cancer Res 76, 2778–2790 (2016). 10.1158/0008-5472.CAN-16-0186 27197267 PMC4874568

[47] Marchetti C. Ovarian cancer onset across different BRCA mutation types: a view to a more tailored approach for BRCA mutated patients. Int J Gynecol Cancer 33, 257–262 (2023). 10.1136/ijgc-2022-003893 36581488

[48] Weigelt B. Diverse BRCA1 and BRCA2 Reversion Mutations in Circulating Cell-Free DNA of Therapy-Resistant Breast or Ovarian Cancer. Clin Cancer Res 23, 6708–6720 (2017). 10.1158/1078-0432.CCR-17-0544 28765325 PMC5728372

[49] Burdett N. L. Multiomic analysis of homologous recombination-deficient end-stage high-grade serous ovarian cancer. Nat Genet 55, 437–450 (2023). 10.1038/s41588-023-01320-2 36849657

[50] Lin K. K. BRCA Reversion Mutations in Circulating Tumor DNA Predict Primary and Acquired Resistance to the PARP Inhibitor Rucaparib in High-Grade Ovarian Carcinoma. Cancer Discov 9, 210–219 (2019). 10.1158/2159-8290.CD-18-0715 30425037

[51] Saner F. A. M. Going to extremes: determinants of extraordinary response and survival in patients with cancer. Nat Rev Cancer 19, 339–348 (2019). 10.1038/s41568-019-0145-5 31076661 PMC7255796

[52] Saner F. A. M. Concurrent RB1 loss and BRCA-deficiency predicts enhanced immunological response and long-term survival in tubo-ovarian high-grade serous carcinoma. medRxiv (2023). 10.1101/2023.11.09.23298321 PMC11325151 38837893

[53] Nelson B. H. Immunological and molecular features of the tumor microenvironment of long-term survivors of ovarian cancer. J Clin Invest 134 (2024). 10.1172/JCI179501 PMC11645148 39470729

[54] Wei L. J. The accelerated failure time model: a useful alternative to the Cox regression model in survival analysis. Stat Med 11, 1871–1879 (1992). 10.1002/sim.4780111409 1480879

[55] Hendry S. Assessing Tumor-Infiltrating Lymphocytes in Solid Tumors: A Practical Review for Pathologists and Proposal for a Standardized Method from the International Immuno-Oncology Biomarkers Working Group: Part 2: TILs in Melanoma, Gastrointestinal Tract Carcinomas, Non-Small Cell Lung Carcinoma and Mesothelioma, Endometrial and Ovarian Carcinomas, Squamous Cell Carcinoma of the Head and Neck, Genitourinary Carcinomas, and Primary Brain Tumors. Adv Anat Pathol 24, 311–335 (2017). 10.1097/PAP.0000000000000161 28777143 PMC5638696

[56] Garsed D. W. Homologous Recombination DNA Repair Pathway Disruption and Retinoblastoma Protein Loss Are Associated with Exceptional Survival in High-Grade Serous Ovarian Cancer. Clin Cancer Res 24, 569–580 (2018). 10.1158/1078-0432.CCR-17-1621 29061645

[57] Wang C. Pooled Clustering of High-Grade Serous Ovarian Cancer Gene Expression Leads to Novel Consensus Subtypes Associated with Survival and Surgical Outcomes. Clin Cancer Res 23, 4077–4085 (2017). 10.1158/1078-0432.CCR-17-0246 28280090 PMC5567822

[58] Patch A. M. Whole-genome characterization of chemoresistant ovarian cancer. Nature 521, 489–494 (2015). 10.1038/nature14410 26017449

[59] Delahunty R. TRACEBACK: Testing of Historical Tubo-Ovarian Cancer Patients for Hereditary Risk Genes as a Cancer Prevention Strategy in Family Members. J Clin Oncol 40, 2036–2047 (2022). 10.1200/JCO.21.02108 35263119 PMC9197360

[60] Nguyen L., Martens W. M., J., Van Hoeck, A. & Cuppen, E. Pan-cancer landscape of homologous recombination deficiency. Nature Communications 11, 1–12 (2020). 10.1038/s41467-020-19406-4 PMC7643118 33149131

[61] Wang J. DNA methylation-based profiling reveals distinct clusters with survival heterogeneity in high-grade serous ovarian cancer. Clin Epigenetics 13, 190 (2021). 10.1186/s13148-021-01178-3 34645493 PMC8515755

[62] Berenjeno I. M. Oncogenic PIK3CA induces centrosome amplification and tolerance to genome doubling. Nat Commun 8, 1773 (2017). 10.1038/s41467-017-02002-4 29170395 PMC5701070

[63] Smith S. A., Easton D. F., Evans D. G. & Ponder B. A. Allele losses in the region 17q12–21 in familial breast and ovarian cancer involve the wild-type chromosome. Nat Genet 2, 128–131 (1992). 10.1038/ng1092-128 1303261

[64] Gudmundsson J. Different tumor types from BRCA2 carriers show wild-type chromosome deletions on 13q12-q13. Cancer Res 55, 4830–4832 (1995). 7585515

[65] Santana Dos Santos E. Value of the loss of heterozygosity to BRCA1 variant classification. NPJ Breast Cancer 8, 9 (2022). 10.1038/s41523-021-00361-2 35039532 PMC8764043

[66] Maxwell K. N. BRCA locus-specific loss of heterozygosity in germline BRCA1 and BRCA2 carriers. Nat Commun 8, 319 (2017). 10.1038/s41467-017-00388-9 28831036 PMC5567274

[67] Ray-Coquard I. Olaparib plus Bevacizumab as First-Line Maintenance in Ovarian Cancer. N Engl J Med 381, 2416–2428 (2019). 10.1056/NEJMoa1911361 31851799

[68] Ray-Coquard I. Olaparib plus bevacizumab first-line maintenance in ovarian cancer: final overall survival results from the PAOLA-1/ENGOT-ov25 trial. Ann Oncol 34, 681–692 (2023). 10.1016/j.annonc.2023.05.005 37211045

[69] Gonzalez-Martin A. Progression-free survival and safety at 3.5years of follow-up: results from the randomised phase 3 PRIMA/ENGOT-OV26/GOG-3012 trial of niraparib maintenance treatment in patients with newly diagnosed ovarian cancer. Eur J Cancer 189, 112908 (2023). 10.1016/j.ejca.2023.04.024 37263896

[70] Gonzalez-Martin A. Niraparib in Patients with Newly Diagnosed Advanced Ovarian Cancer. N Engl J Med 381, 2391–2402 (2019). 10.1056/NEJMoa1910962 31562799

[71] Gonzalez-Martin A. Maintenance olaparib plus bevacizumab in patients with newly diagnosed advanced high-grade ovarian cancer: Main analysis of second progression-free survival in the phase III PAOLA-1/ENGOT-ov25 trial. Eur J Cancer 174, 221–231 (2022). 10.1016/j.ejca.2022.07.022 36067615

[72] Takaya H., Nakai H., Takamatsu S., Mandai M. & Matsumura N. Homologous recombination deficiency status-based classification of high-grade serous ovarian carcinoma. Sci Rep 10, 2757 (2020). 10.1038/s41598-020-59671-3 32066851 PMC7026096

[73] Subramanian A. Gene set enrichment analysis: a knowledge-based approach for interpreting genome-wide expression profiles. Proc Natl Acad Sci U S A 102, 15545–15550 (2005). 10.1073/pnas.0506580102 16199517 PMC1239896

[74] Castro F., Cardoso A. P., Goncalves R. M., Serre K. & Oliveira M. J. Interferon-Gamma at the Crossroads of Tumor Immune Surveillance or Evasion. Front Immunol 9, 847 (2018). 10.3389/fimmu.2018.00847 29780381 PMC5945880

[75] Newman A. M. Determining cell type abundance and expression from bulk tissues with digital cytometry. Nat Biotechnol 37, 773–782 (2019). 10.1038/s41587-019-0114-2 31061481 PMC6610714

[76] Shi T. Survival Benefit of Germline BRCA Mutation is Associated with Residual Disease in Ovarian Cancer. Cell Physiol Biochem 47, 2088–2096 (2018). 10.1159/000491477 29975922

[77] Harter P. Efficacy of maintenance olaparib plus bevacizumab according to clinical risk in patients with newly diagnosed, advanced ovarian cancer in the phase III PAOLA-1/ENGOT-ov25 trial. Gynecol Oncol 164, 254–264 (2022). 10.1016/j.ygyno.2021.12.016 34952708

[78] Marchetti C. BRCA Mutation Status to Personalize Management of Recurrent Ovarian Cancer: A Multicenter Study. Ann Surg Oncol 25, 3701–3708 (2018). 10.1245/s10434-018-6700-6 30128899

[79] Bercow A. Utilization of Primary Cytoreductive Surgery for Advanced-Stage Ovarian Cancer. JAMA Netw Open 7, e2439893 (2024). 10.1001/jamanetworkopen.2024.39893 39412808 PMC11581664

[80] Ponzone R. BRCA1/2 status and chemotherapy response score to tailor ovarian cancer surgery. Crit Rev Oncol Hematol 157, 103128 (2021). 10.1016/j.critrevonc.2020.103128 33137578

[81] Telli M. L. Homologous Recombination Deficiency (HRD) Score Predicts Response to Platinum-Containing Neoadjuvant Chemotherapy in Patients with Triple-Negative Breast Cancer. Clin Cancer Res 22, 3764–3773 (2016). 10.1158/1078-0432.CCR-15-2477 26957554 PMC6773427

[82] Webb J. R., Milne K. & Nelson B. H. PD-1 and CD103 Are Widely Coexpressed on Prognostically Favorable Intraepithelial CD8 T Cells in Human Ovarian Cancer. Cancer Immunol Res 3, 926–935 (2015). 10.1158/2326-6066.CIR-14-0239 25957117

[83] Gersekowski K. Germline BRCA variants, lifestyle and ovarian cancer survival. Gynecol Oncol 165, 437–445 (2022). 10.1016/j.ygyno.2022.03.020 35400525 PMC9133192

[84] Philpott C., Tovell H., Frayling I. M., Cooper D. N. & Upadhyaya M. The NF1 somatic mutational landscape in sporadic human cancers. Hum Genomics 11, 13 (2017). 10.1186/s40246-017-0109-3 28637487 PMC5480124

[85] Kobel M. Survey of NF1 inactivation by surrogate immunohistochemistry in ovarian carcinomas. Gynecol Oncol 178, 80–88 (2023). 10.1016/j.ygyno.2023.09.016 37820398

[86] Schroder J., Corbin V. & Papenfuss A. T. HYSYS: have you swapped your samples? Bioinformatics 33, 596–598 (2017). 10.1093/bioinformatics/btw685 28003257 PMC5408803

[87] Song S. qpure: A tool to estimate tumor cellularity from genome-wide single-nucleotide polymorphism profiles. PLoS One 7, e45835 (2012). 10.1371/journal.pone.0045835 23049875 PMC3457972

[88] Van Loo P. Allele-specific copy number analysis of tumors. Proc Natl Acad Sci U S A 107, 16910–16915 (2010). 10.1073/pnas.1009843107 20837533 PMC2947907

[89] Wingett S. W. & Andrews S. FastQ Screen: A tool for multi-genome mapping and quality control. F1000Res 7, 1338 (2018). 10.12688/f1000research.15931.2 30254741 PMC6124377

[90] Li H. & Durbin R. Fast and accurate short read alignment with Burrows-Wheeler transform. Bioinformatics 25, 1754–1760 (2009). 10.1093/bioinformatics/btp324 19451168 PMC2705234

[91] Shen R. & Seshan V. E. FACETS: allele-specific copy number and clonal heterogeneity analysis tool for high-throughput DNA sequencing. Nucleic Acids Res 44, e131 (2016). 10.1093/nar/gkw520 27270079 PMC5027494

[92] Dobin A. STAR: ultrafast universal RNA-seq aligner. Bioinformatics 29, 15–21 (2013). 10.1093/bioinformatics/bts635 23104886 PMC3530905

[93] Anders S., Pyl P. T. & Huber W. HTSeq--a Python framework to work with high-throughput sequencing data. Bioinformatics 31, 166–169 (2015). 10.1093/bioinformatics/btu638 25260700 PMC4287950

[94] Robinson M. D., McCarthy D. J. & Smyth G. K. edgeR: a Bioconductor package for differential expression analysis of digital gene expression data. Bioinformatics 26, 139–140 (2010). 10.1093/bioinformatics/btp616 19910308 PMC2796818

[95] Ritchie M. E. limma powers differential expression analyses for RNA-sequencing and microarray studies. Nucleic Acids Res 43, e47 (2015). 10.1093/nar/gkv007 25605792 PMC4402510

[96] Aryee M. J. Minfi: a flexible and comprehensive Bioconductor package for the analysis of Infinium DNA methylation microarrays. Bioinformatics 30, 1363–1369 (2014). 10.1093/bioinformatics/btu049 24478339 PMC4016708

[97] Laumont C. M. Single-cell Profiles and Prognostic Impact of Tumor-Infiltrating Lymphocytes Coexpressing CD39, CD103, and PD-1 in Ovarian Cancer. Clin Cancer Res 27, 4089–4100 (2021). 10.1158/1078-0432.CCR-20-4394 33963000

[98] Millstein J. Prognostic gene expression signature for high-grade serous ovarian cancer. Annals of Oncology 31, 1240–1250 (2020). 10.1016/j.annonc.2020.05.019 32473302 PMC7484370

[99] Talhouk A. Development and Validation of the Gene Expression Predictor of High-grade Serous Ovarian Carcinoma Molecular SubTYPE (PrOTYPE). Clinical Cancer Research 26, 5411–5423 (2020). 10.1158/1078-0432.CCR-20-0103 32554541 PMC7572656

[100] Lai Z. VarDict: a novel and versatile variant caller for next-generation sequencing in cancer research. Nucleic Acids Res 44, e108 (2016). 10.1093/nar/gkw227 27060149 PMC4914105

[101] McKenna A. The Genome Analysis Toolkit: a MapReduce framework for analyzing next-generation DNA sequencing data. Genome Res 20, 1297–1303 (2010). 10.1101/gr.107524.110 20644199 PMC2928508

[102] Kim S. Strelka2: fast and accurate calling of germline and somatic variants. Nat Methods 15, 591–594 (2018). 10.1038/s41592-018-0051-x 30013048

[103] Koboldt D. C. VarScan 2: somatic mutation and copy number alteration discovery in cancer by exome sequencing. Genome Res 22, 568–576 (2012). 10.1101/gr.129684.111 22300766 PMC3290792

[104] Li H. The Sequence Alignment/Map format and SAMtools. Bioinformatics 25, 2078–2079 (2009). 10.1093/bioinformatics/btp352 19505943 PMC2723002

[105] Tan A., Abecasis G. R. & Kang H. M. Unified representation of genetic variants. Bioinformatics 31, 2202–2204 (2015). 10.1093/bioinformatics/btv112 25701572 PMC4481842

[106] Van den Eynden J., Fierro A. C., Verbeke L. P. & Marchal K. SomInaClust: detection of cancer genes based on somatic mutation patterns of inactivation and clustering. BMC Bioinformatics 16, 125 (2015). 10.1186/s12859-015-0555-7 25903787 PMC4410004

[107] Chen X. Manta: rapid detection of structural variants and indels for germline and cancer sequencing applications. Bioinformatics 32, 1220–1222 (2016). 10.1093/bioinformatics/btv710 26647377

[108] Cameron D. L. GRIDSS: sensitive and specific genomic rearrangement detection using positional de Bruijn graph assembly. Genome Res 27, 2050–2060 (2017). 10.1101/gr.222109.117 29097403 PMC5741059

[109] Wala J. A. SvABA: genome-wide detection of structural variants and indels by local assembly. Genome Res 28, 581–591 (2018). 10.1101/gr.221028.117 29535149 PMC5880247

[110] Nariai N. HLA-VBSeq: accurate HLA typing at full resolution from whole-genome sequencing data. BMC Genomics 16 Suppl 2, S7 (2015). 10.1186/1471-2164-16-S2-S7 PMC4331721 25708870

[111] Hundal J. pVACtools: A Computational Toolkit to Identify and Visualize Cancer Neoantigens. Cancer Immunol Res 8, 409–420 (2020). 10.1158/2326-6066.CIR-19-0401 31907209 PMC7056579

[112] Sztupinszki Z. Migrating the SNP array-based homologous recombination deficiency measures to next generation sequencing data of breast cancer. NPJ Breast Cancer 4, 16 (2018). 10.1038/s41523-018-0066-6 29978035 PMC6028448

[113] Love M. I., Huber W. & Anders S. Moderated estimation of fold change and dispersion for RNA-seq data with DESeq2. Genome Biol 15, 550 (2014). 10.1186/s13059-014-0550-8 25516281 PMC4302049

[114] Wilkerson M. D. & Hayes D. N. ConsensusClusterPlus: a class discovery tool with confidence assessments and item tracking. Bioinformatics 26, 1572–1573 (2010). 10.1093/bioinformatics/btq170 20427518 PMC2881355

[115] Harrell F. E. Regression modeling strategies (2nd ed.). Springer International Publishing (2016). 10.1007/978-3-319-19425-7

[116] Grambsch P. M. & Therneau T. M. Proportional Hazards Tests and Diagnostics Based on Weighted Residuals. Biometrika 81, 515–526 (1994).

[117] Team, R. C. R: A language and environment for statistical computing. (2023).

